# Ferulic acid in combination with ginsenoside Rb1 alleviates myocardial no-reflow by inhibiting platelet HMGB1 release and NET formation

**DOI:** 10.1186/s13020-025-01303-x

**Published:** 2026-01-08

**Authors:** Jia Li, Yue You, Yilin Wang, Jialu Zou, Shunli Xiao, Xiaojie Yin, Jing Xu, Fulong Liao, Huamin Zhang, Yun You

**Affiliations:** 1 State Key Laboratory for Quality Ensurance and Sustainable Use of Dao-Di Herbs, Institute of Chinese Materia Medica, China Academy of Chinese Medical Sciences, Beijing, 100700 China; 2 Institute of Basic Theory for Chinese Medicine, China Academy of Chinese Medical Sciences, Beijing, 100700 China; 3 School of Pharmaceutical Sciences, Hunan University of Medicine, Huaihua, 418000 China

**Keywords:** Ferulic acid, Ginsenoside Rb1, Neutrophil extracellular traps, Platelets, High mobility group box-1, Myocardial no-reflow

## Abstract

**Background:**

The no-reflow (NR) phenomenon remains a challenge in the treatment of acute myocardial infarction. This study aimed to explore the therapeutic potential and underlying mechanism of a combination of ferulic acid (FA) and ginsenoside Rb1 (Rb1), active components of the traditional Chinese herbal pair of *Ligusticum chuanxiong* Hort. and *Panax ginseng* C. A. Mey., respectively, in alleviating myocardial ischemia–reperfusion injury (MIRI) and NR.

**Methods:**

A rat model of MIRI was established to evaluate the effects of FA and Rb1 on cardiac function, infarction/NR area, microthrombi formation, and serum biomarkers. An integrated strategy combining network pharmacology, molecular docking, and molecular dynamics simulations was employed to predict key pathways and targets. Platelet HMGB1 release and neutrophil extracellular trap (NET) formation were investigated both in vitro and vivo.

**Results:**

MIRI induced obvious NR, accompanied by enhanced platelet HMGB1 release, increased NET formation and microthrombi accumulation. Bioinformatical analyses confirmed that FA and Rb1 stably interacts with HMGB1 and PAD4. Experimentally, FA predominantly inhibited platelet HMGB1 release, with IC_50_ of 19.28 µM, by suppressing the p38/ERK1/2 pathway. Rb1 exhibited stronger efficacy in inhibiting PAD4 enzyme activity. The FA-Rb1 combination demonstrated superior effects compared to either agent alone, effectively suppressing NET formation, improving cardiac function, and reducing both NR area and microthrombi burden.

**Conclusions:**

The combination of FA and Rb1 not only inhibits platelet HMGB1 release but also reduces NETs, thereby enhancing anti-NR efficacy. These findings propose a novel therapeutic approach involving FA-Rb1 combination therapy for alleviating myocardial NR.

**Graphical Abstract:**

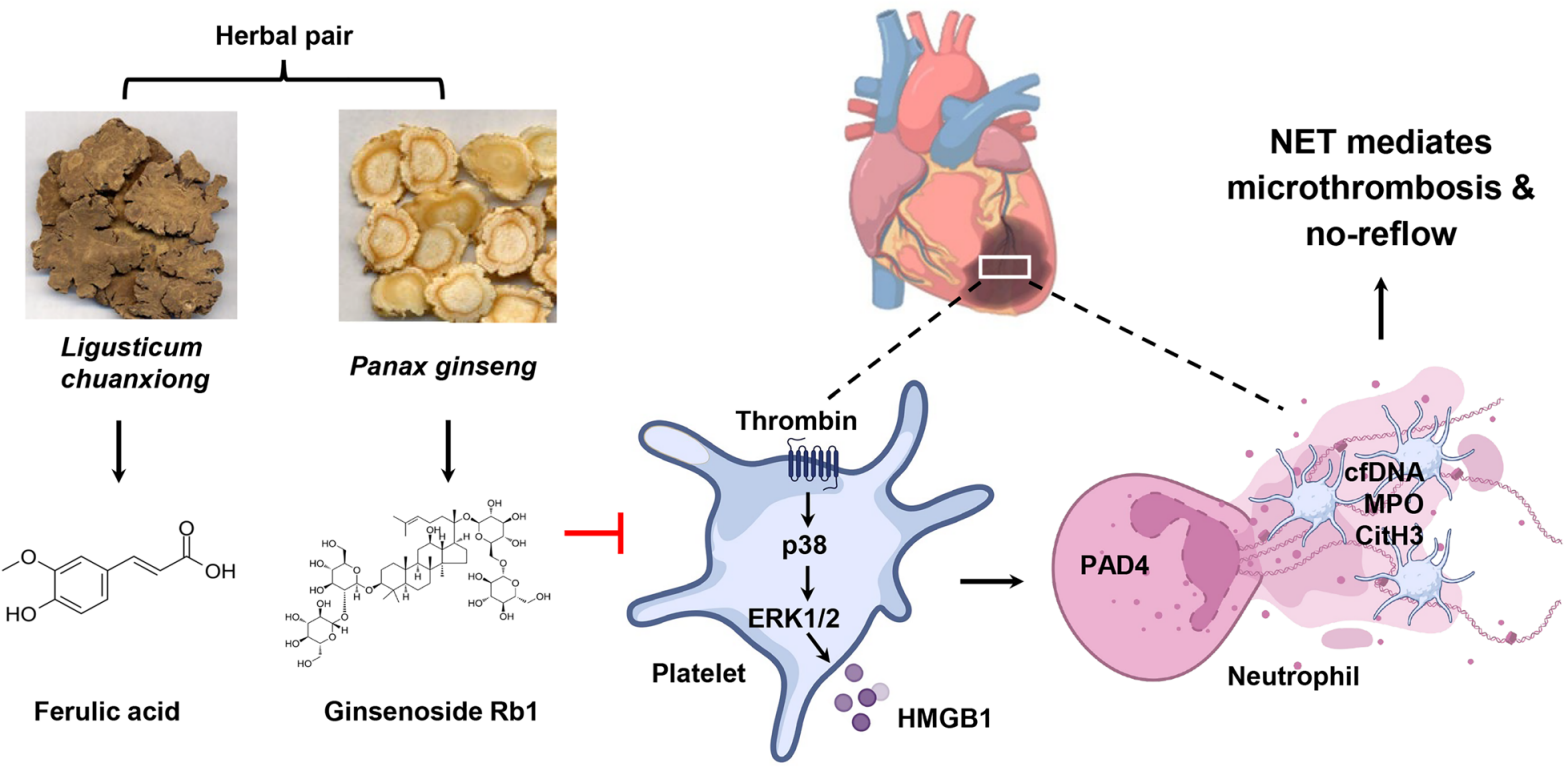

**Supplementary Information:**

The online version contains supplementary material available at 10.1186/s13020-025-01303-x.

## Introduction

Acute myocardial infarction (AMI) continues to be a major contributor to morbidity and mortality [1]. The no-reflow (NR) phenomenon, characterized by inadequate tissue perfusion after successful reopening of the infarct-related coronary artery, occurs in up to 50% of AMI cases [2]. This phenomenon compromises the benefits of reperfusion therapy, leading to worsened prognosis and an increased risk of major adverse cardiovascular events [3]. The pathogenesis of NR is multifactorial, with microvascular obstruction being the most prominent underlying mechanism [4]. NR results from microvascular obstruction caused by spasm, microthrombi, and reperfusion injury, coupled with neutrophil/platelet accumulation and neurohumoral system activation [5]. Current pharmacologic management primarily use vasodilators and antiplatelet agents [6]. However, a significant proportion of NR cases are refractory to available treatments [7], highlighting the urgent need for novel therapeutic strategies.

Following AMI, neutrophils are the first inflammatory cells to infiltrate the ischemic myocardium [8]. They release pro-inflammatory mediators and form neutrophil extracellular traps (NETs), which are DNA-based structures that promote coagulation by scaffolding procoagulant molecules [9]. Growing evidence indicates that NETs play pivotal roles in the pathophysiology of cardiovascular diseases [10]. For instance, NET-mediated microthrombosis contributes to myocardial NR in myocardial I/R injury [11, 12]. Moreover, in patients with coronary heart disease, elevated levels of circulating cell-free DNA and nucleosomes, markers of NET formation, are correlated with a prothrombotic state and adverse cardiac outcomes [13]. Thus, targeting NET formation represents a promising therapeutic approach for NR.

Myocardial infarction triggers sterile inflammation. Endothelial injury leads to exposure of adhesive proteins and subsequent platelet activation. Activated platelets release damage-associated molecular patterns such as high-mobility group box 1 (HMGB1), which promotes thrombosis and facilitates NET formation [14]. HMGB1 is a highly conserved nuclear protein that functions as a chromatin-binding factor regulating DNA architecture and gene transcription. When released extracellularly during stress or injury, HMGB1 acts as a damage-associated molecular pattern molecule, triggering inflammatory and immune responses involved in various pathological conditions such as sepsis, ischemia–reperfusion injury, and cancer [15, 16]. In myocardial ischemia–reperfusion injury (MIRI), HMGB1 functions as an early mediator of inflammation and organ damage [17]. Primarily released from inflammatory stimulus-activated monocytes or macrophages, it triggers the NF-κB transcriptional network via the TLR4-dependent signaling pathway, thereby amplifying the inflammatory response as a pathological cascade that culminates in histopathological myocardial injury [18]. NR is driven by a vicious cycle: excessive platelet activation triggers massive HMGB1 release, which binds to receptor for advanced glycation end products (RAGE) on neutrophils to induce NET formation [19]; NETs then further promote platelet aggregation and microthrombosis, ultimately causing microvascular obstruction [11, 14]. Studies using megakaryocyte/platelet-specific HMGB1-deficient mice have demonstrated attenuated immunothrombosis, underscoring its critical role in thrombotic processes [20, 21]. In human coronary artery thrombi, HMGB1 is associated with adverse clinical outcomes [22]. Protein arginine deiminase 4 (PAD4), an enzyme predominantly expressed in neutrophils, mediates chromatin decondensation during NETosis [23]. Accordingly, a combinatorial pharmacological approach involving the inhibition of HMGB1 release from platelets and NET formation has significant clinical implications.

The “Yiqi Huoxue” formula is widely applied in clinical practice for the management of cardiovascular diseases based on Traditional Chinese Medicine principles, which emphasize Qi supplementation and blood circulation activation. *Panax ginseng* C. A. Mey. (ginseng) and *Ligusticum chuanxiong* Hort. (Chuanxiong) are the primary constituent herbs within this formula, with ginseng serving to supplement Qi and Chuanxiong promoting blood circulation. These two herbs are often administered together to enhance their individual therapeutic effects [24, 25]. This pair is documented in the “Dictionary of Chinese Medicine Prescription” with a usage frequency of 23.31% [26], and its active compounds are widely applied in cardiovascular diseases treatment [27, 28]. Ginsenoside Rb1 (Rb1), the main active component in ginseng, has been reported to protect against MIRI [29, 30]. Our previous work furture demonstrated that Rb1 significantly inhibits calcium influx in platelets [31]. Ferulic acid (FA), the primary active component of Chuanxiong, exhibits multiple pharmacological properties, including inhibition of platelet aggregation [32, 33] and anti-inflammatory effects [34]. In this study, we integrated network pharmacology, molecular docking, molecular dynamics (MD) simulations, and both in vitro and in vivo experimental validation to investigate the effects of FA and Rb1, with the aim of revealing the pharmacological mechanism of this Traditional Chinese Medicine (TCM) herbal pair. We demonstrated for the first time, that the combination of FA and Rb1 significantly attenuates NR and infarct size in a rat model of MIRI.

## Material and methods

### Reagents

FA (B20007), Rb1 (B21050), and glycyrrhizin (B20417) (purity ≥ 98% by HPLC) were purchased from Shanghai Yuanye Bio-Technology Co., Ltd (Shanghai, China). DNase I (11,284,932,001) was purchased from Sigma-Aldrich (St Louis, MO, USA). Creatine kinase-MB (CK-MB, ml059533) and cardiac troponin I (cTnI, ml059111) ELISA kits were obtained from Mlbio (Shanghai, China). HMGB-1 (E-EL-R0505) ELISA kit was bought from Elabscience (Wuhan, China), while CitH3 (ER2113) ELISA kit was obtained from Fine Biotech Co., Ltd. (Wuhan, China). Hoechst 33,342 was acquired from Invitrogen (Cleveland, MA, USA), prostaglandin E1 (HY-B0131) from MedChemExpress (New Jersey, USA), and thrombin (T832140-1KU) from Macklin (Shanghai, China). Thioflavin S dye (T1892) and 2,3,5-triphenyltetrazolium chloride monohydrate (TTC, T8877) were bought from Sigma-Aldrich (St Louis, MO, USA). SB203580 (HY-10256), U0126 (HY-12031A), and anisomycin (HY-18982) were purchased from MedChemExpress (New Jersey, USA). Percoll (17–0891-01) was purchased from Cytiva (Logan, USA), giemsa Staining Solution (C0131) was purchased from Beyotime (Shanghai, China), and PAD4 Inhibitor Screening Assay Kit (700,560) from Cayman Chemical (Ann Arbor, MI, USA).

The following antibodies were used in this study: rabbit anti-CD41 (ab181582, Abcam, Cambridge, UK), mouse anti-myeloperoxidase (ab90810, Abcam, Cambridge, UK), rabbit anti-histone H3 (ab281584, Abcam, Cambridge, UK), FITC-conjugated anti-CD61 (104,305, BioLegend, California, USA), PE-conjugated anti-HMGB1 (651,404, BioLegend, California, USA), rabbit anti-p-p38 (4511, Cell Signaling Technology, Massachusetts, USA), rabbit anti-p38 (8690, Cell Signaling Technology, Massachusetts, USA), rabbit anti-p-ERK1/2 (4370, Cell Signaling Technology, Massachusetts, USA), rabbit anti-ERK1/2 (4695, Cell Signaling Technology, Massachusetts, USA), rabbit anti-HMGB1 (ab79823, Abcam, Cambridge, UK), rabbit anti-GAPDH (10,494–1-AP, Proteintech, Wuhan, China) and rabbit anti-β-actin (81,115–1-RR, Proteintech, Wuhan, China).

### Animals

Adult male Sprague–Dawley rats (180–220 g) were purchased from SPF Biotechnology Co., Ltd (Beijing, China), with an animal production license (Certificate No.: SCXK [Jing] 2023–0077). All animal care and experimental procedures were approved by the Ethics Committee of the Institute of Laboratory Animal Center Institute of Basic Theory for Chinese Medicine, China Academy of Chinese Medical Sciences, under ethics approval number IBTCMCACMS21-2404–03. The certificate number of the experimental facility is SYXK (Jing) 2021–0017. The rats were maintained in a stable environment (22 ± 1 °C, 55%–65% humidity, 12-h light/dark cycle) and were given food and water freely.

### Rat model of myocardial I/R

Rats were randomized to receive prophylactic intraperitoneal injections of glycyrrhizic acid (GA group) at a dose of 50 mg·kg^−1^ day^−1^ or DNase I at a dose of 5 mg·kg^−1^ day^−1^ (6 days and 1 h before myocardial I/R). The dosage and route of administration were selected based on previous studies [35, 36]. The myocardial I/R rat model was established as previously described [37]. The rats were anesthetized with 1% pentobarbital (50 mg·kg^−1^), with respiration maintained using a ventilator. The left chest was opened between the third and fourth ribs to expose the heart. Myocardial ischemia was induced by ligating the left anterior descending (LAD) coronary artery with a 6–0 silk suture for 45 min, followed by reperfusion for 24 h after suture removal. Blood and cardiac tissues were then collected for subsequent analysis. In sham controls, the LAD was threaded but not ligated. Rats were randomized to receive prophylactic intraperitoneal injections of FA (50 mg·kg^−1^ day^−1^), Rb1 (50 mg·kg^−1^ day^−1^), or FA plus Rb1 (both at 50 mg·kg^−1^ day^−1^) (6 days and 1 h before myocardial I/R). Sodium ferulate, a derivative of ferulic acid, is used in clinic for the treatment of cardiovascular diseases and the prevention of thrombosis [38]. The clinical intravenous dose of sodium ferulate is 0.3 g·day^−1^. Based on a human-to-rat equivalent dose ratio, the FA dose of 50 mg·kg^−1^ day^−1^ used in this study was twice that of the clinical equivalent. Rb1 reduced the infarct size in MIRI from range of 20 ~ 80 mg·kg^−1^ day^−1^ [39], the Rb1 dose of 50 mg·kg^−1^ day^−1^ was chosen in the subsequent studies. The sham and I/R groups were injected intraperitoneally with saline as controls.

### Ultrasound assessment of cardiac function in rats

Echocardiographic analysis was conducted using a Vevo 3100 ultra-high-resolution small animal ultrasound imaging system (VisualSonics, Toronto, Ontario, Canada). After applying a coupling agent to the chest and upper abdomen of the rats, M-mode and B-mode ultrasound images were obtained under the guidance of a two-dimensional probe. Ejection fraction (EF) and fractional shortening (FS) were used to evaluate cardiac function. Each parameter was calculated as the mean value of three consecutive cardiac cycles.

### Real-time myocardial contrast echocardiography (MCE)

MCE was performed 24 h post-reperfusion employing the Vevo 3100 imaging system. Following anesthesia, the rats were placed in the supine position, and imaging commenced once the cardiac rhythm stabilized. A high-frequency transducer was used for anatomical localization, followed by M-mode echocardiographic acquisition in the left ventricular long-axis view. A long-lasting microbubble solution (18 μL·mL^−1^, supervue-MB, Nanjing Leapsonics Medical Technology Co., Ltd, China) was administered via tail vein injection (0.2 mL per rat) under constant infusion. Myocardial contrast perfusion videos were recorded for subsequent analysis of time-to-peak enhancement.

### Assessment of infarcted myocardial areas and NR

Post-reperfusion, 4% thioflavin S (1 mL·kg^−1^) was administered via the inferior vena cava. After 1 min of circulation in the dark, the rats were euthanized. Collected cardiac tissues were rinsed with saline to remove surface blood, snap-frozen at − 80 °C for 5 min, and embedded in a cardiac mold. Subsequently, the heart tissue located beneath the ligature line was meticulously divided into five slices, each ~ 2 mm thick. Tissue sections were first visualized under 365-nm UV illumination for the delineation of the NR area, followed by 2% TTC staining for 10 min at room temperature in darkness to demarcate the infarct area. ImageJ software was used to quantify the NR, infarcted, and total myocardial areas.

### Analysis of HMGB1 expression on platelet surfaces and neutrophil-platelet aggregates

Blood samples collected via abdominal aorta puncture were anticoagulated with 3.8% sodium citrate (1:9) and diluted 1:10 with DPBS. Samples were then incubated for 30 min at room temperature in the dark with the following antibody combinations: FITC-anti-CD61 (1:50) and PE-anti-HMGB1 (1:20) to assess platelet surface HMGB1; FITC-anti-CD61 (1:50) and PE-anti-CD11b/c (1:80) to detect neutrophil-platelet aggregates (NPAs). After incubation, samples were fixed in 1% paraformaldehyde and then quantified by flow cytometry (BD FACS Symphony A1, BD Biosciences, CA, USA).

### Enzyme-linked immunosorbent assay (ELISA)

The serum levels of cTnI and CK-MB, as well as the plasma or cell supernatant levels of HMGB1 and CitH3, were measured using the respective ELISA kits according to the manufacturers’ protocols. Absorbance at 450 nm was measured on a Multiskan microplate reader (Thermo Fisher Scientific, Waltham, MA, USA).

### Histological and immunohistochemical staining

The heart was excised and preserved in 4% paraformaldehyde. Tissues embedded in paraffin were deparaffinized using xylene and examined through hematoxylin and eosin (HE) staining. For immunohistochemical staining, the tissue sections were also deparaffinized with xylene, followed by the inactivation of endogenous peroxidase activity. Antigen retrieval was carried out using citrate buffer. The tissues were then blocked with 10% goat serum for 1 h at room temperature and subsequently incubated overnight at 4 °C with a rabbit anti-CD41 antibody (1:500). Tissues were treated with a reaction enhancer (PV-9001, ZSBio, China) for 20 min at 37 °C, followed by incubation with HRP-conjugated secondary antibody for 1 h at room temperature. Subsequently, tissues were stained with diaminobenzidine and counterstained with haematoxylin. Finally, images were observed and photographed with a Nikon ECLIPSE Ni-U optical microscope (Tokyo, Japan).

### NET imaging

For immunofluorescence staining of heart tissue, sections were deparaffinized, rehydrated, and subjected to antigen retrieval in sodium citrate buffer (pH 6). After blocking with 10% goat serum, sections were incubated with primary antibodies against CitH3 (1:500) and MPO (1:25) at room temperature for 1 h, followed by an overnight incubation at 4 °C. A fluorescent secondary antibody (1:200) was subsequently added dropwise, avoiding light. Nuclei were counterstained with Hoechst 33,342, and images were captured using an Abberior Stedycon confocal microscope (Abberior Instruments GmbH, Gottingen, Germany).

For immunofluorescence staining of cells, platelets were first pretreated for 10 min with either Rb1 or FA and then with 0.5 U·mL^−1^ thrombin for 30 min. To induce NET formation, the platelets were then co-cultured with neutrophils adhered to poly-L-lysine-coated coverslips at a 150:1 ratio for 2 h at 37 °C with 5% CO_2_. After stimulation, unbound cells were washed away, while adhered cells were fixed in 4% paraformaldehyde, blocked with 5% goat serum, and incubated with primary antibodies against CitH3 (1:2000) and MPO (1:50) initially at room temperature for 1 h, and then overnight at 4 °C in the dark. After washing, the cells were incubated with fluorescent secondary antibodies (1:200) and then counterstained with Hoechst 33,342 for 10 min. Subsequently, images were captured using the Abberior Stedycon confocal microscope. NET quantification was performed referring to the method described in Xia et al. [40]. Neutrophils and NET-forming neutrophils were identified based on MPO and extracellular CitH3 positivity, respectively. The percentage of NET-forming neutrophils was calculated as (number of CitH3 + cells / number of MPO + cells) × 100. Bliss independence model [41] was applied to assess the synergistic effect of FA and Rb1 in NET formation. The combination index (CI) value was calculated as follows: CI = (E_FA_ + E_Rb1_—E_FA_ × E_Rb1_)/ E_FA+Rb1_. A CI value less than 1 indicates the synergistic effects of FA and Rb1.

### Network pharmacology

The targets of FA and Rb1 were predicted using the SwissTargetPrediction online tool (http://swisstargetprediction.ch/), the PharmMapper server (http://lilab-ecust.cn/pharmmapper/), and the BATMAN-TCM database (http://bionet.ncpsb.org.cn/batman-tcm/). The GeneCards (https://www.genecards.org/) and OMIM (https://omim.org/) databases were used to search for targets related to acute coronary syndrome (ACS). Additionally, the GSE61144 chip dataset [42] was downloaded from Gene Expression Omnibus (GEO, https://www.ncbi.nlm.nih.gov/geo/), which includes samples from 10 normal individuals and 14 patients with ACS. After normalization of the expressed genes using the “limma” package [43] in R, differentially expressed genes (DEGs) were screened using P ≤ 0.05 and |logFC|≥ 0.5 as the criteria. The STRING database (https://cn.string-db.org) was used to predict interactions between the target genes. The R packages “clusterProfiler” and “enrichplot” [44] were employed for Gene Ontology (GO) term and Kyoto Encyclopedia of Genes and Genomes (KEGG) pathway enrichment analysis of the DEGs. Bar charts for GO and KEGG enrichment results (significance threshold: P < 0.05) were generated.

### Immune infiltration analysis

A quantitative analysis of the relative levels of infiltrating immune cells in 24 samples from the GSE61144 dataset was undertaken using the CIBERSORT deconvolution algorithm [45]. Furthermore, the correlation between the expression levels of core genes and immune cell infiltration was explored based on Spearman’s correlation coefficient (P < 0.05).

### Molecular docking

The 2D structures (SDF format) of FA and Rb1 as ligands were downloaded from the PubChem database (https://pubchem.ncbi.nlm.nih.gov/) and Open Babel 2.3.2 software was used to convert the SDF files into PDB files. The three-dimensional crystal structure (PDB format) of HMGB1 (PDB ID: 4QR9) and PAD4 (PDB ID: 1WDA) was downloaded from the PDB protein database (https://www.rcsb.org/) for use as the receptor protein. Receptor proteins were prepared in PYMOL 2.3.4 by removing water and ligands, and in AutoDockTools by adding hydrogens and balancing charges, with both receptors and ligands converted to PDBQT format. Molecular docking was then performed using AutoDock Vina 1.1.2 to identify the optimal conformation with the lowest binding free energy, and the results were visualized with PYMOL.

### Molecular dynamics simulation

MD simulations of FA and Rb1 bound to HMGB1 and PAD4 were performed using Gromacs2022.3 software [46]. Small molecules were prepared with the GAFF force field (AmberTools22), hydrogenation, and RESP charge calculation (Gaussian 16W) [47]. Simulations were performed at 300 K and 1 bar using the Amber99sb-ildn force field, Tip3p water model, and neutralization with Na⁺ ions [48]. The system underwent energy minimization (steepest descent), followed by 100 ps equilibration in both NVT and NPT ensembles (100,000 steps each; coupling constant: 0.1 ps). A final 100 ns production MD (5,000,000 steps, 2-fs step length) was conducted. Trajectories were analyzed for RMSD, RMSF, SASA, RG, hydrogen bonds, and free energy topography.

### Preparation of washed platelets from rats

SD rats were anesthetized with 1% pentobarbital sodium (50 mg·kg^−1^, i.p.), and blood was collected from the abdominal aorta using 3.8% sodium citrate as an anticoagulant. Platelet-rich plasma (PRP) was then obtained by centrifuging the anticoagulated blood at 200 × g for 10 min. Acid Citrate Dextrose (ACD) and ethylenediaminetetraacetic acid (EDTA) were added to the PRP at a ratio of 9:1:0.2, respectively, followed by the addition of prostaglandin E1 (5 µM). The mixture was then centrifuged at 400 × g for 5 min, following which the platelet pellets were resuspended in Tyrode’s solution (138 mM sodium chloride, 3.3 mM sodium dihydrogen phosphate, 2.9 mM potassium chloride, 1 mM magnesium chloride, 1 mM calcium chloride, 5.5 mM glucose, 20 mM HEPES, pH 7.4), ACD, and EDTA at a ratio of 9:1:0.2, and then centrifuged at 400 × g for 5 min at room temperature to remove plasma proteins. The platelet pellets were resuspended in Tyrode’s solution, yielding washed platelets.

### Flow cytometric detection of HMGB1 expression in thrombin-stimulated platelets

Washed platelets from rats were adjusted to a density of 3 × 10^8^ cells/mL using a calcium-containing buffer. Different concentrations of FA and Rb1 (25, 50, 100, and 200 μM) were added to the platelets, followed by incubation at room temperature for 10 min. Thrombin (0.5 U·mL ^−1^) was then added to induce platelet activation for 30 min at room temperature. Subsequently, FITC- anti-CD61 (1:50) and PE-anti-HMGB1 (1:20) were separately added to 100 μL of the washed platelets, followed by gentle mixing and incubation in the dark at room temperature for 30 min. The cells were then fixed in 1% paraformaldehyde and the expression of HMGB1 on the platelet surface was analyzed by flow cytometry.

### Western blot

Platelets were pretreated with FA (20 μM) and GA (10 μM), then incubated with thrombin (0.05 U·mL ^−1^) for 10 min. To explore whether p38/ERK1/2/HMGB1 pathway in platelets were regulated by FA, washed platelets also were preincubated with SB203580 (an inhibitor of p38), U0126 (an inhibitor of ERK1/2), anisomycin (an activator of p38) and FA + anisomycin. Total proteins of platelets were extracted by RIPA buffer and quantified using a BCA kit (Beyotime Biotechnology, Shanghai, China). Equal amounts of platelet proteins were separated by 12% SDS-PAGE and transferred to PVDF membranes. Subsequently, the membrane was blocked using a 5% BSA solution and incubated overnight at 4 °C with primary antibodies: p-p38 (1:1000), p38 (1:1000), p-ERK1/2 (1:2000), ERK1/2 (1:1000), GAPDH (1:10,000), HMGB1 (1:1000), β-actin (1:50,000). Following incubation with HRP-conjugated secondary antibodies (1:5000), chemiluminescence detection was performed on the membranes. The band intensities were quantified using ImageJ software.

### Extraction of neutrophils from the peripheral blood of rats

Whole blood was collected from the abdominal aorta of rats using 3.8% sodium citrate as an anticoagulant. The diluted blood was layered at the interface of 81% and 62% Percoll and then centrifuged at 710 × g for 30 min. Cells at the 62%/81% interface was collected. The contaminating red blood cells were lysed followed by washing with PBS. The pellets containing the neutrophils were then resuspended in RPMI 1640 medium supplemented with 5% FBS. Neutrophil purity reached up to 90% (Figure S1A).

### PAD4 inhibitor assay

The inhibitory capacities of different concentrations of FA and Rb1 (25, 50, 100, and 200 μM) were determined using a PAD4 inhibitor Screening Assay Kit according to the manufacturer’s instructions. Measurements were made with a SpectraMax i3x fluorometric microplate reader (Molecular Devices, USA) (excitation/emission = 410/475 nm).

### Statistical analysis

All data, presented as means ± SE from independent biological replicates, were analyzed blindly using GraphPad Prism 9.0. Normality and homogeneity of variance were assessed using the Shapiro–Wilk test. Accordingly, comparisons between two groups used the independent samples t-test, while multi-group comparisons employed one-way ANOVA or the nonparametric Kruskal–Wallis test for non-normally distributed data. The half-maximal inhibitory concentration (IC_50_) was determined by normalizing data to vehicle controls and applying nonlinear regression. Significance was set at P < 0.05.

## Results

### Platelet HMGB1 release and NET formation contribute to myocardial no-reflow following I/R injury in rats

**Fig. 1:**
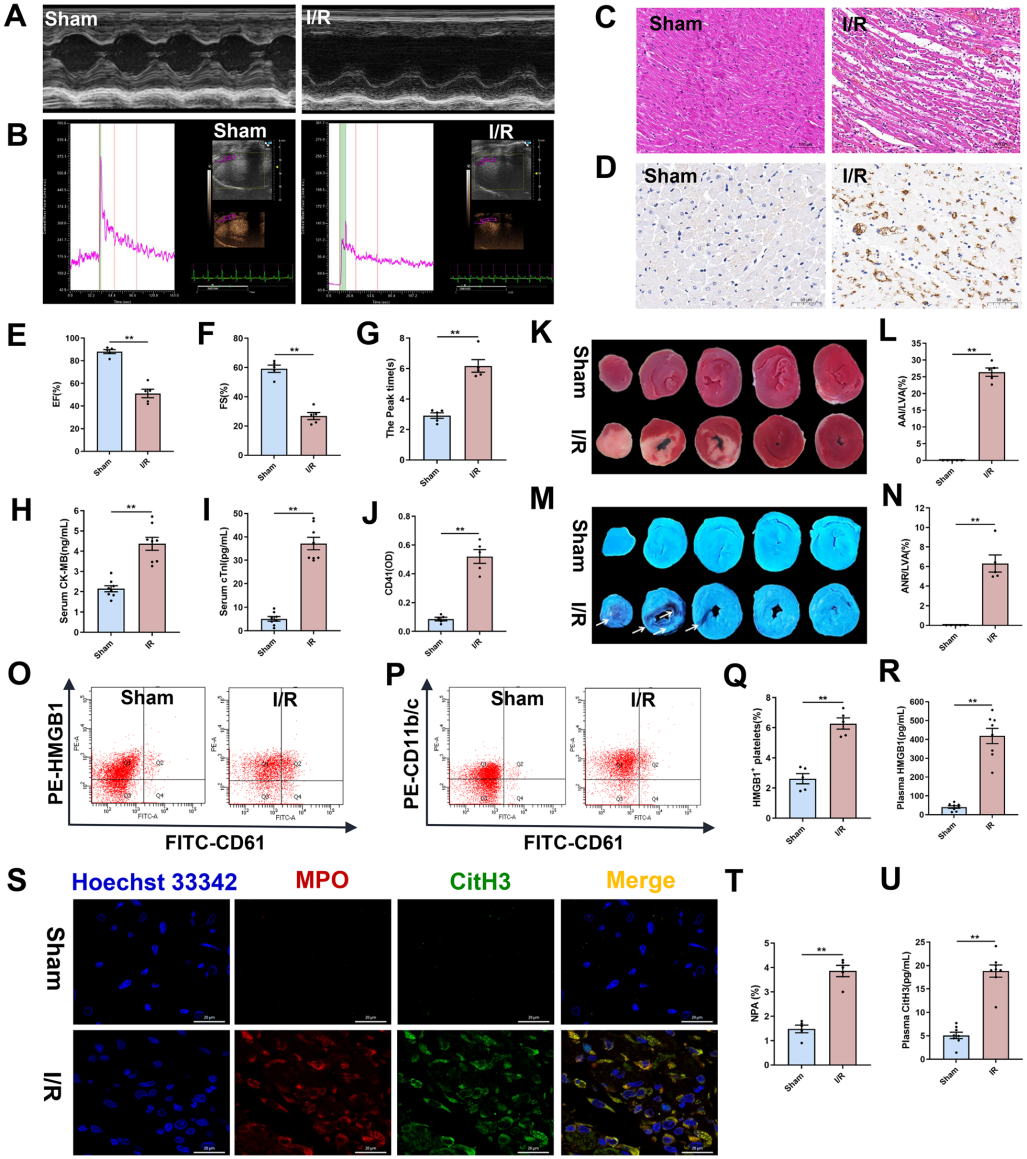
Platelet HMGB1 release and NET formation contribute to myocardial no-reflow following I/R Injury in rats. Rats were subjected to 45 min of LAD followed by 24 h of reperfusion or sham surgery. **A** Representative images of echocardiography. **B** Representative images of real-time MCE. **C** Representative images of HE staining in heart sections (scale bar: 100 μm). **D** Representative images of immunohistochemical staining for CD41 in heart sections (scale bar: 50 μm). **E**, **F** Quantitative analysis of left ventricular EF and FS (n = 5). **G** Values of myocardial perfusion peak time (n = 5). (**H** and **I**) Serum CK-MB and cTnI levels were measured by ELISA (n = 8). **J** Quantitative analysis of CD41 (n = 5). **K** TTC staining. **L** Quantitative analysis of myocardial infarction area (n = 5). **M** Thioflavin S staining. **N** Quantitative analysis of NR area (n = 5). **O**, **P** Representative flow cytometry scatter plot of platelet HMGB1 expression and NPA levels in the diluted whole blood. **Q** Quantitative analysis of platelet surface HMGB1. **R** Plasma HMGB1 levels was measured by ELISA (n = 8). **S** Representative immunofluorescence images of NET formation stained with MPO (red), CitH3 (green), and Hoechst 33,342 (blue) (scale bar: 20 μm). **T** Quantitative analysis of NPA levels (n = 5). **U** Plasma CitH3 levels was measured by ELISA (n = 8). Data are presented as means ± SE. Significance is indicated by the lines and symbols above the graphs for pairwise comparison (**P < 0.01)

A rat model of I/R injury was established by subjecting the animals to 45 min of ischemia followed by 24 h of reperfusion. Transesophageal echocardiography revealed that, compared to the sham group, rats in the I/R model group exhibited significant reductions in EF and FS, indicating severe impairment of cardiac function and structure (Fig. 1A, E, F). Given that myocardial infarction is closely associated with inadequate blood supply, we assessed regional myocardial blood flow using MCE. Real-time MCE showed a significant prolongation of the peak myocardial perfusion time in the model group compared to the sham group (Fig. 1B, G). Consistent with this observation, I/R injury resulted in a myocardial infarct area to approximately 26.4 ± 1.2% (Fig. 1K, L) and a NR area to about 6.3 ± 0.9% as assessed by TTC staining and Thioflavin S staining respectively (Fig. 1M, N). Serum levels of the cardiac injury markers CK-MB and cTnI were also markedly elevated in the model group (Fig. 1H, I). HE staining showed more inflammatory cell infiltration in the myocardial tissues of the model group (Fig. 1C). Immunohistochemical staining for CD41, a marker for microthrombi, was performed on heart sections following I/R injury, which revealed a marked increase in microthrombus formation in the model group (Fig. 1D, J).

The release of HMGB1 following myocardial injury initiates a pathogenic cascade by activating neutrophils and platelets. This activation, in turn, prompts further HMGB1 release from platelets, which amplifies the process by promoting microthrombi and facilitating NETosis [49]. Substantiating this cascade, we observed a marked increase in the circulating NPAs proportion (Fig. 1P, T), and a dramatic elevation in plasma HMGB1, from 40.5 ± 6.8 ng·L^−1^ in sham group to 418.6 ± 40.3 ng·L^−1^ in the model group (Fig. 1R). This was accompanied by a twofold increase in HMGB1 expression on platelet membranes (Fig. 1O, Q). Consistent with enhanced NETosis, immunofluorescence staining revealed stronger expression of MPO and CitH3 in the ischemic myocardium (Fig. 1S), a finding further supported by significantly elevated plasma CitH3 levels as determined by ELISA (Fig. 1U). Collectively, these results underscore a cycle of HMGB1 driven inflammation, NETosis and mirothrombi that propagates myocardial damage and NR.

### The inhibition of HMGB1 expression and NET formation alleviated NR in rats

**Fig. 2:**
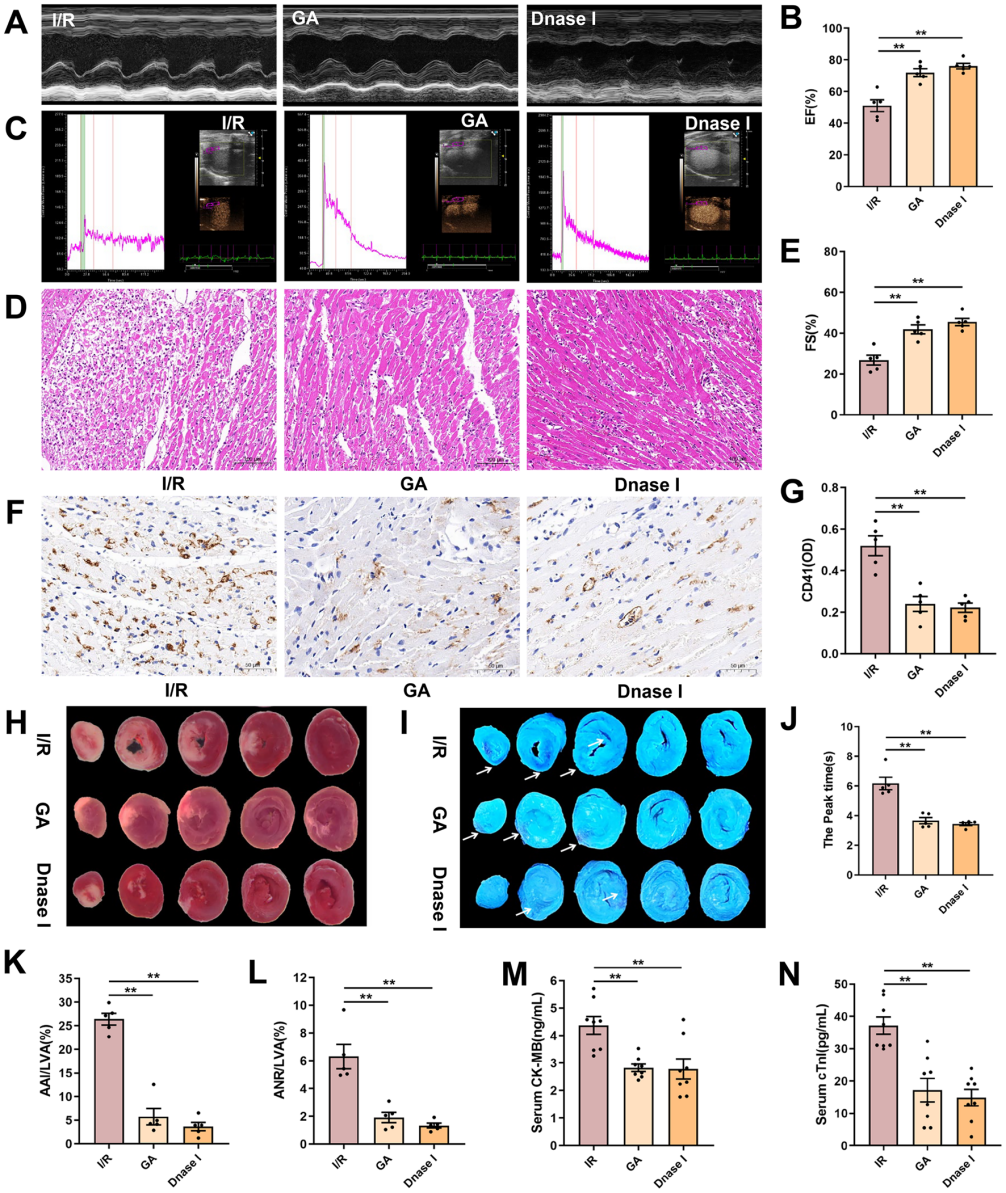
GA and DNase I alleviate NR. Rats were subjected to 45 min of LAD followed by 24 h of reperfusion or sham surgery. Rats were prophylactically administered with saline, GA (50 mg·kg^−1^ day^−1^) or DNase I (5 mg·kg^−1^ day^−1^). **A** Representative images of echocardiography. **B**, **E** Quantitative analysis of left ventricular EF and FS (n = 5). **C** Representative images of real-time MCE. **D** Representative images of HE staining in heart sections (scale bar: 100 μm). **F** Representative images of immunohistochemical staining for CD41 in heart sections (scale bar: 50 μm). **G** Quantitative analysis of CD41 (n = 5). **H** TTC staining. **I** Thioflavin S staining. **J** Values of myocardial perfusion peak time (n = 5). **K** Quantitative analysis of myocardial infarction area (n = 5). **L** Quantitative analysis of NR area (n = 5). **M, N** Serum CK-MB and cTnI levels were measured by ELISA (n = 8). Data are presented as means ± SE. Significance is indicated by the lines and symbols above the graphs for pairwise comparison (**P < 0.01)

GA, a small-molecule inhibitor of HMGB1 [50] and DNase I an FDA-approved endonuclease that degrades NETs and cfDNA [51] were used to investigate the role of the HMGB1 and NETs in MIRI and NR phenomenon. Both interventions significantly improved cardiac function, as indicated by transesophageal echocardiography showing elevated EF and FS (Fig. 2A, B, E). Real-time MCE further revealed that GA and DNase I shortened the myocardial perfusion peak time compared to the model group (Fig. 2C, J). Histological and immunohistochemical analyses demonstrated that GA and DNase I alleviated I/R-induced pathological changes. HE staining showed reduced inflammatory cell infiltration (Fig. 2D), and immunohistochemistry confirmed that both treatments attenuated microthrombi formation (Fig. 2F, G). Furthermore, GA and DNase I significantly reduced myocardial infarct size by 78.3 ± 4.4% and 86.3 ± 4.3%, respectively (Fig. 2H, K), and diminished the NR area as assessed by thioflavin S staining, with reductions of 69.6 ± 15.2% (GA) and 78.8% ± 14.9% (DNase I) (Fig. 2I, L). Consistently, both treatments also suppressed the I/R induced elevations in serum CK-MB and cTnI levels (Fig. 2M, N).

**Fig. 3:**
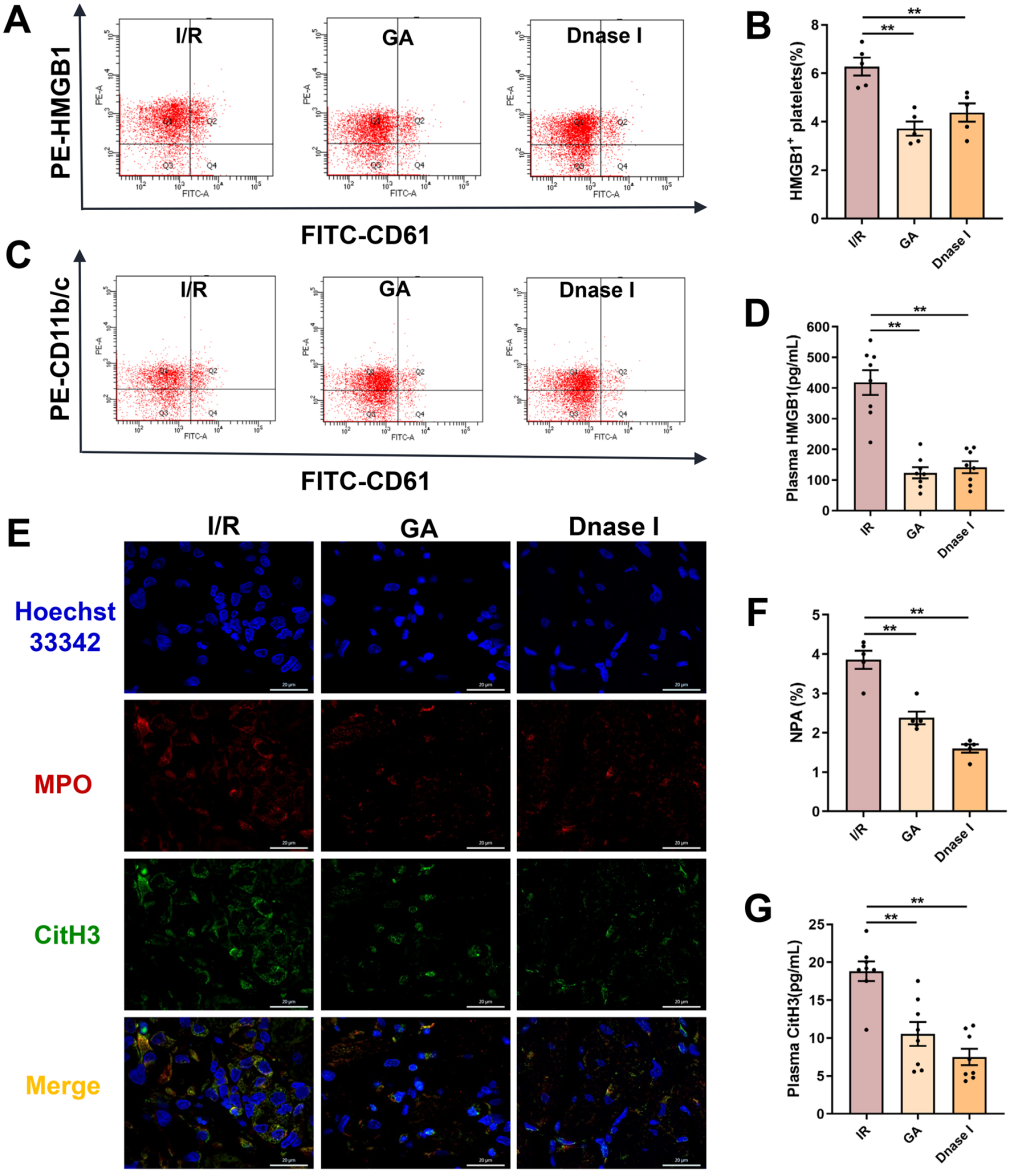
The inhibition of HMGB1 expression and NET formation alleviated NR phenomenon in rats. Rats were subjected to 45 min of LAD followed by 24 h of reperfusion or sham surgery. Rats were prophylactically administered with saline, GA (50 mg·kg^−1^ day^−1^) or DNase I (5 mg·kg^−1^ day^−1^). **A**, **C** Representative flow cytometry scatter plot of platelet HMGB1 expression and NPA levels in the diluted whole blood. **B** Quantitative analysis of platelet surface HMGB1. **D** Plasma HMGB1 levels was measured by ELISA (n = 8). **E** Representative immunofluorescence images of NET formation stained with MPO (red), CitH3 (green), and Hoechst 33,342 (blue) (scale bar: 20 μm). **F** Quantitative analysis of NPA levels (n = 5). **G** Plasma CitH3 levels was measured by ELISA (n = 8). Data are presented as means ± SE. Significance is indicated by the lines and symbols above the graphs for pairwise comparison (**P < 0.01)

At the molecular level, GA and DNase I significantly reduced HMGB1 expression on platelets with inhibition rate of 40.8 ± 10.9% and 30.3 ± 11.9%, respectively (Fig. 3A, B), and suppressed plasma HMGB1 levels by 70.4 ± 1.5% and 66.1 ± 1.6% (Fig. 3D). Consistent with this attenuation of HMGB1 signaling, the proportion of NPAs in peripheral blood was markedly decreased with GA and DNase I treatment (Fig. 3C, F). Immunofluorescence staining further revealed that both agents effectively attenuated NET formation in the myocardium, as shown by diminished expression of MPO and CitH3 (Fig. 3E). Correspondingly, plasma CitH3 levels were significantly reduced by GA and DNase I, with inhibition rate of 44.0 ± 7.1% and 60.1 ± 6.4% respectively (Fig. 3G). Taken together, these findings indicates that HMGB1 and NETosis cascade, are the important therapeutic targets for alleviating MIRI and NR phenomenon.

### Immune cell infiltration analysis predicted that the expression of PAD4 is elevated in patients with ACS and is strongly correlated with neutrophil levels

**Fig. 4:**
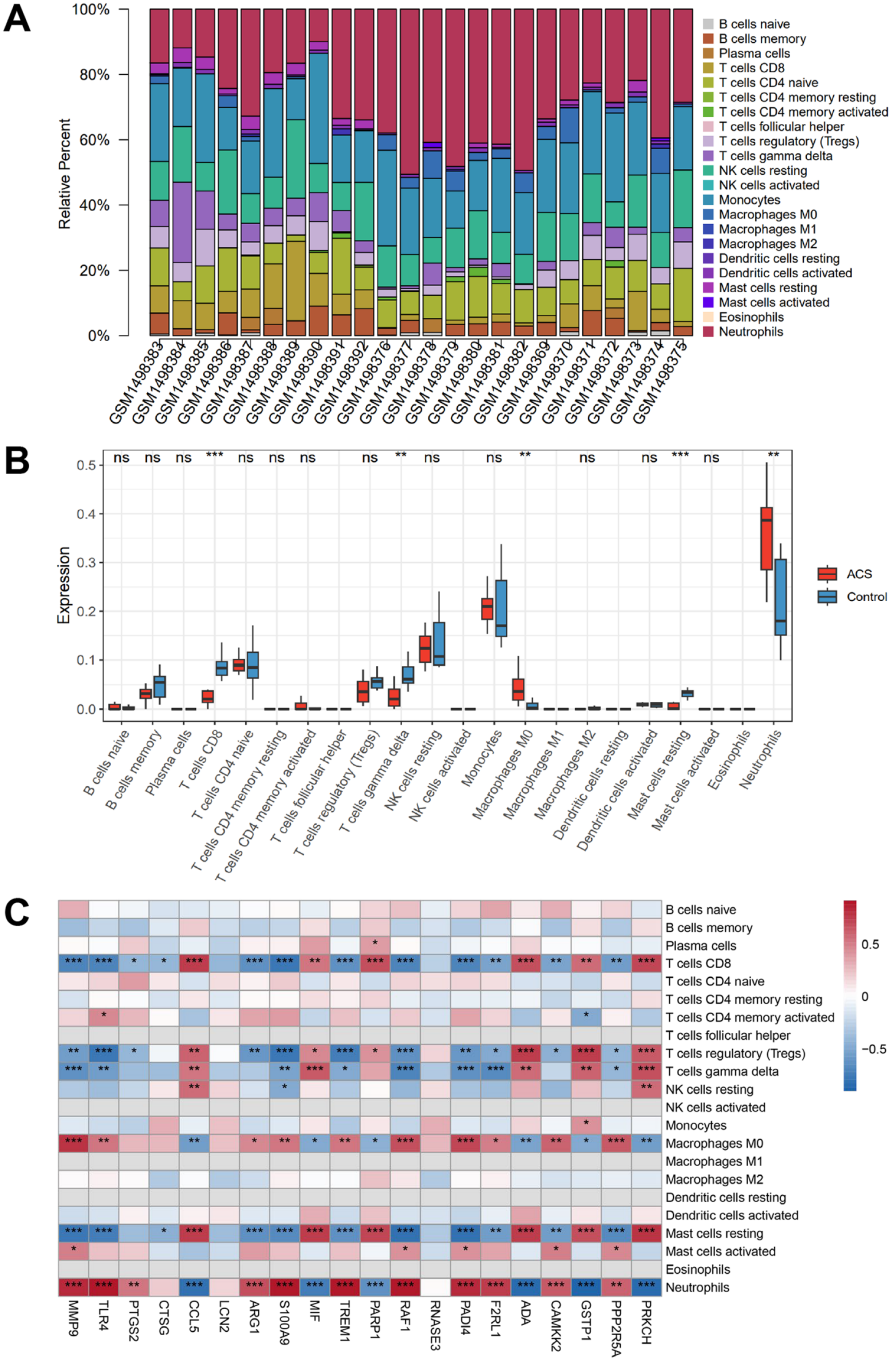
Immune cell infiltration analysis predicted that the expression of PAD4 is elevated in patients with ACS and is strongly correlated with neutrophil levels. **A** Stacked histogram of immune cell proportions in samples. The horizontal axis represents sample numbers, and the bars are color-coded to indicate different types of immune cells, with the area of each color block representing the proportion of that immune cell in the overall infiltration. **B** Comparison of immune infiltration scores between normal and ACS samples. The blue and red bar charts represent normal and ACS patients, respectively, with the height of the bars reflecting the immune cell infiltration scores for each group (**p < 0.01 and ***p < 0.001). **C** Heatmap of the correlation between key targets and immune cells. Blue indicates a negative correlation (r < 0), while red indicates a positive correlation (r > 0), with the intensity of the color being proportional to the absolute value of the correlation coefficient (*p < 0.05, **p < 0.01, and ***p < 0.001)

Immune cell infiltration analysis indicated a significant upregulation of PAD4 expression in patients with ACS, which showed a strong positive correlation with neutrophil levels. Neutrophils exhibited the highest infiltration rate in the peripheral blood of ACS patients (Fig. 4A). The immune-inflammatory response is activated during myocardial infarction, characterized by an increased proportion of neutrophils and M0 macrophages, alongside a decreased proportion of CD8 + T cells, gamma delta T cells, and resting mast cells (Fig. 4B). Among the 22 key DEGs identified between control and ACS groups, PAD4, MMP9, TLR4, CCL5, and S100A9 exhibited strong correlations with the infiltration levels of neutrophils, M0 macrophages, CD8 + T cells, gamma delta T cells, and resting mast cells, respectively. These findings suggested that these key genes may contribute to ACS progression by modulating the infiltration of specific immune cell subpopulations. A correlation heatmap depicting the relationships between key targets and immune cell scores is presented in Fig. 4C.

### Network pharmacology predicted that FA in combination with Rb1 inhibits NET formation by targeting PAD4

**Fig. 5:**
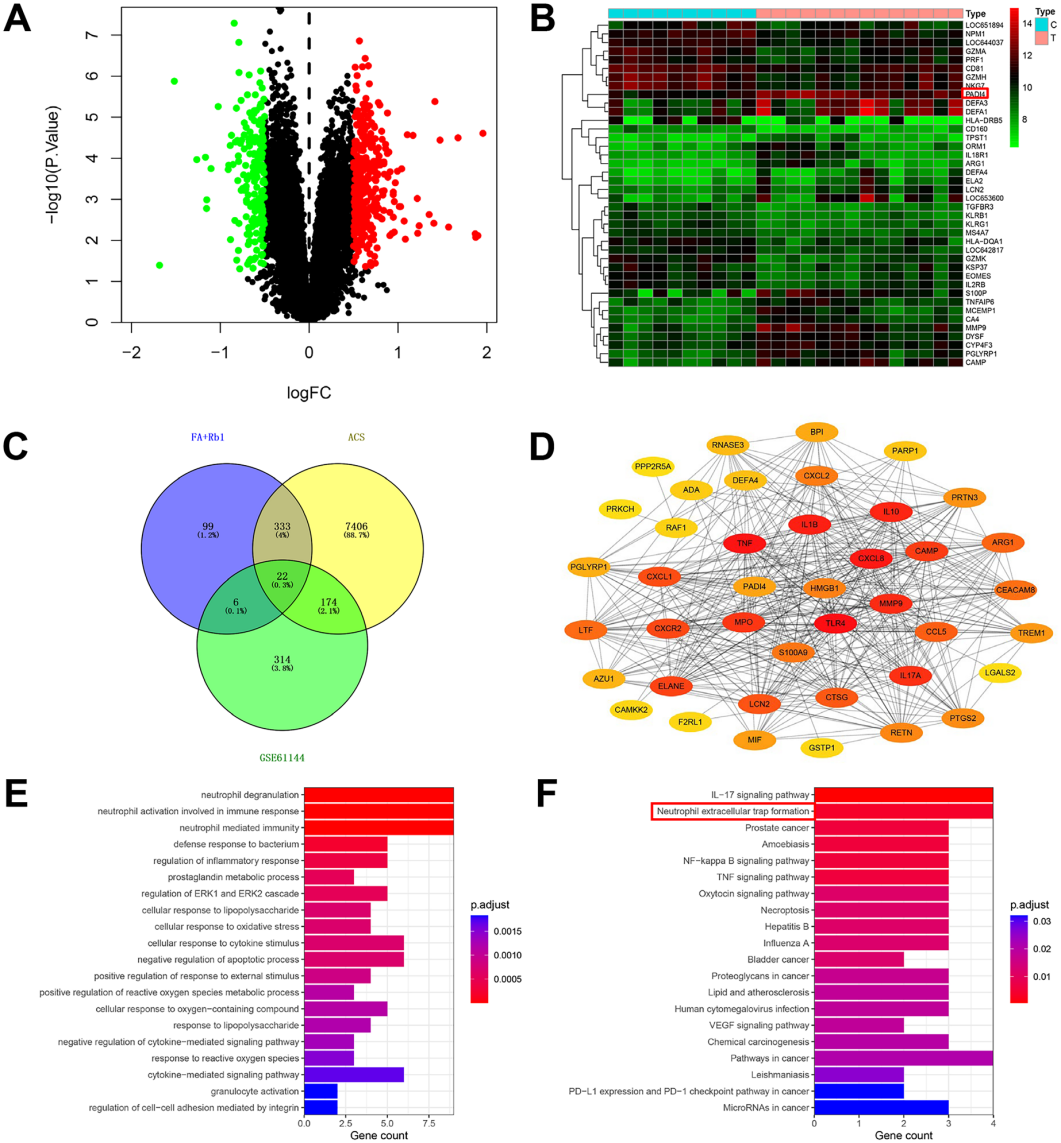
Network pharmacology predicted that FA in combination with Rb1 inhibits NET formation by targeting PAD4. **A** Volcano plot: Red and green represent significantly upregulated and downregulated genes in ACS, respectively, while non-differential genes are shown in black. **B** Heatmap of differential genes (Top 20 |logFC|). Red indicates upregulation, and green indicates downregulation. **C** Venn diagram of active components of FA in combination with Rb1 and ACS disease targets. **D** PPI protein interaction network. **E** GO biological process enrichment analysis results. **F** KEGG pathway enrichment analysis results

Given that FA and Rb1 are recognized active compounds in Chuanxiong and Ginseng, respectively, network pharmacology was utilized to investigate the potential biological mechanisms and target genes of the FA-Rb1 combination in ACS treatment. Analysis of the GSE61144 dataset identified 516 DEGs, comprising 326 upregulated and 190 downregulated genes. A volcano plot of these DEGs (Fig. 5A) and a list of the top 20 upregulated and downregulated genes ranked by |logFC| are shown in Fig. 5B. A total of 7,935 ACS-related targets were retrieved from the GeneCards and OMIM databases. Using SwissTargetPrediction, PharmMapper, and BATMAN-TCM, 460 potential targets of the FA-Rb1 combination were identified. Intersection analysis yielded 22 common targets between the FA-Rb1 combination and ACS (Fig. 5C). A Protein–Protein Interaction (PPI) network for these 22 targets was constructed using the STRING database (confidence score > 0.4), with the number of first-level interactors limited to 20. Visualization of the PPI network in Cytoscape 3.7.2 highlighted key targets such as HMGB1 and PAD4 (Fig. 5D). GO biological process enrichment analysis of the DEGs revealed significant involvement in pathways including neutrophil degranulation, neutrophil activation involved in immune response, and neutrophil-mediated immunity (Fig. 5E). KEGG pathway enrichment analysis indicated involvement of neutrophil extracellular trap formation and several inflammatory signaling pathways (e.g., IL-17, NF-κB, TNF) in ACS (Fig. 5F). These results suggested that the identified key targets may promote ACS progression by modulating immune and inflammatory pathways.

### FA and Rb1 displayed good affinity and stability with HMGB1 and PAD4

**Fig. 6:**
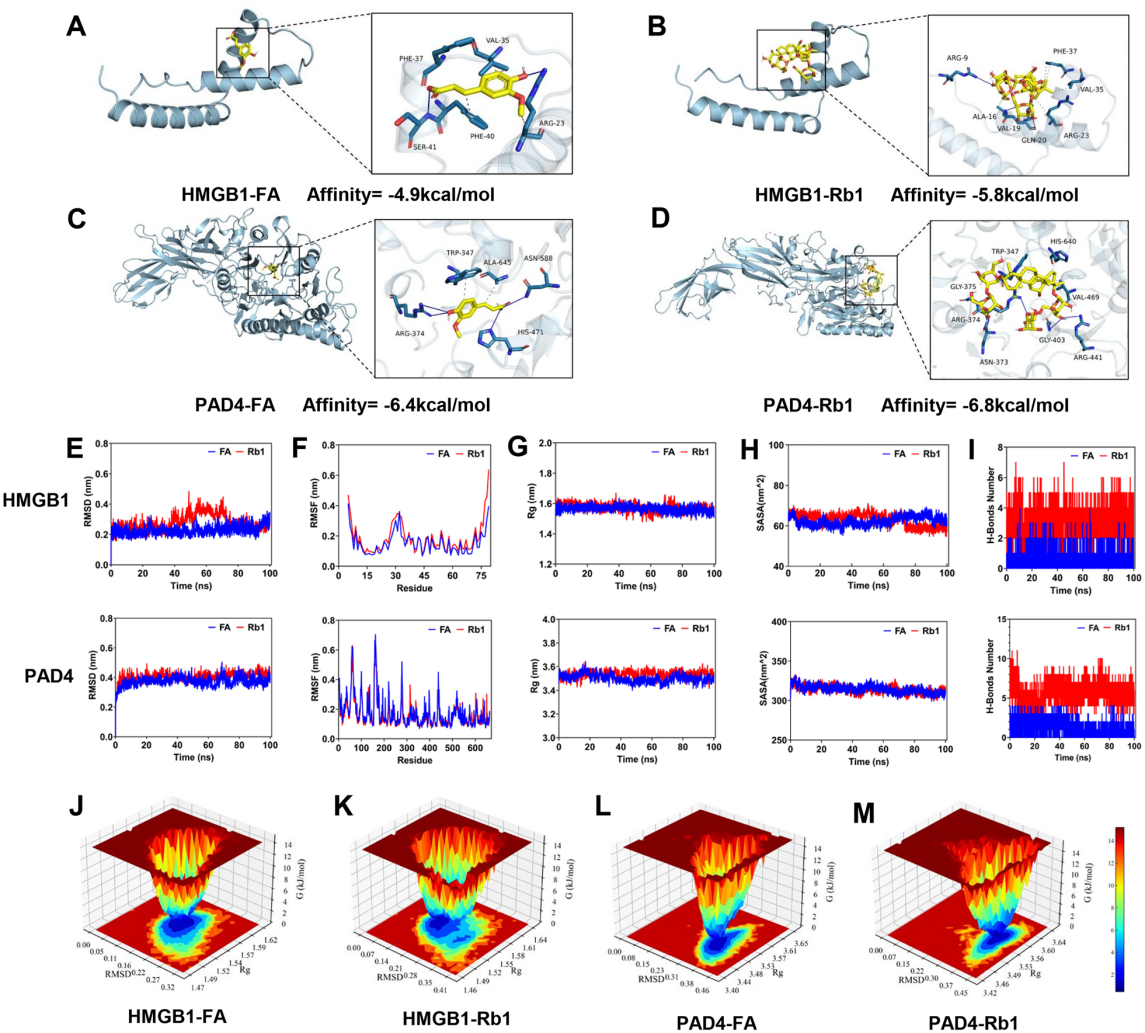
FA and Rb1 displayed good binding affinity and stability with HMGB1 and PAD4. **A** Molecular docking patterns of FA with HMGB1. **B** Molecular docking patterns of Rb1 with HMGB1. **C** Molecular docking patterns of FA with PAD4. **D** Molecular docking patterns of Rb1 with PAD4. The blue solid lines represent hydrogen bonds formed between each ligand and the target amino acid residues. **E** RMSD curve. **F** RMSF curve. **G** Rg curve. **H** SASA curve. **I** Fluctuation curve of the number of hydrogen bonds. **J** Free energy landscape of FA with HMGB1. **K** Free energy landscape of Rb1 with HMGB1. **L** Free energy landscape of FA with PAD4. **M** Free energy landscape of Rb1 with PAD4

Molecular docking demonstrated favorable binding conformations for both FA and Rb1 with HMGB1 and PAD4. The calculated binding free energies were − 4.9 kcal/mol (FA-HMGB1), − 6.4 kcal/mol (FA-PAD4), − 5.8 kcal/mol (Rb1-HMGB1), and − 6.8 kcal/mol (Rb1-PAD4), indicating good binding affinity. Analysis of the complexes (Fig. 6A-D) revealed that FA forms hydrogen bonds with HMGB1 (residues ARG23, SER41) and PAD4 (residues ARG374, HIS471, ASN588), whereas Rb1 forms hydrogen bonds with HMGB1 (residues ARG9, GLN20) and PAD4 (residues ASN373, ARG374, GLY375, GLY403, ARG441). MD simulations were performed to validate binding stability. All systems reached equilibrium, as evidenced by stable RMSD values fluctuating between 0.2 and 0.5 nm (Fig. 6E). Low RMSF values (0.1–0.4 nm) indicated high residue stability (Fig. 6F). The complexes maintained structural compactness, with stable Rg values approximating 1.50–1.65 nm for HMGB1-FA/Rb1 and 3.45–3.60 nm for PAD4-FA/Rb1 (Fig. 6G). Consistent SASA values (HMGB1-FA: ~ 62 nm^2^; HMGB1-Rb1: ~ 64 nm^2^; PAD4-FA: ~ 312 nm^2^; PAD4-Rb1: ~ 315 nm^2^) confirmed stable binding interfaces (Fig. 6H). Hydrogen bond analysis revealed persistent interactions: HMGB1-FA (1–2 bonds), HMGB1-Rb1 (3–5 bonds), PAD4-FA (2–3 bonds), and PAD4-Rb1 (5–8 bonds) (Fig. 6I). Free energy landscapes displayed singular, smooth minimum energy clusters, confirming that the complexes resided in highly stable conformational states (Fig. 6J-M). Collectively, these MD simulations demonstrate that FA and Rb1 form stable, high-affinity complexes with HMGB1 and PAD4.

### FA predominately inhibited platelet HMGB1 release and Rb1 mainly suppressed PAD4 activity

**Fig. 7:**
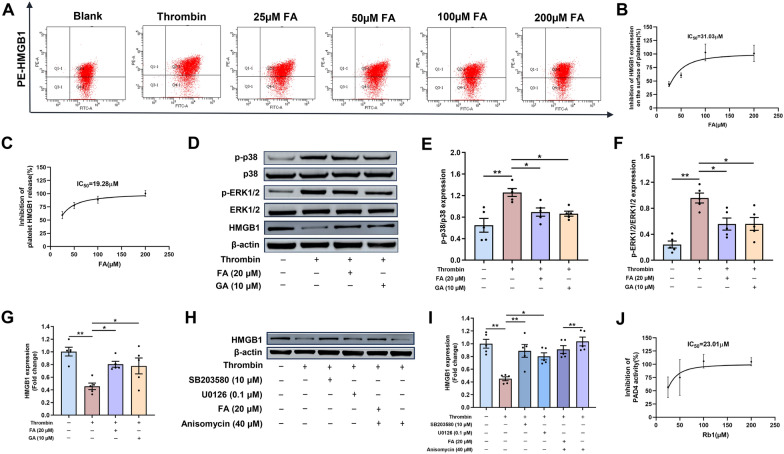
FA predominately inhibited platelet HMGB1 release and Rb1 mainly suppressed PAD4 activity. **A** Representative flow cytometry scatter plot of platelet HMGB1 expression on washed platelets isolated from rats. **B** The effect of FA on thrombin-induced HMGB1 expression on the surface of platelets (n = 3). **C** HMGB1 levels in conditioned media derived from resting and activated platelets were also measured by ELISA (n = 5). **D** Platelets were pretreated with FA (20 μM) and GA (10 μM), then incubated with thrombin (0.05U·mL^−1^) for 10 min. Subsequently, immunoblotting was performed. **E**–**G** Quantitative analysis of p-p38/p38, p-ERK1/2/ ERK1/2 and HMGB1/β-actin (n = 5). **H** Platelets were pretreated with SB203580 (an inhibitor of p38), U0126 (an inhibitor of ERK1/2), anisomycin (an activator of p38), and FA + anisomycin, and then incubated with thrombin (0.05U·mL^−1^) for 10 min. Subsequently, immunoblotting was performed. **I** Quantitative analysis of HMGB1/β-actin (n = 5). **J** Enzymatic activity of PAD4 was measured in a cell-free assay upon co-incubation of FA (25, 50, 100, and 200 μM) with the PAD4-substrate (n = 5). Data are presented as means ± SE. Significance is indicated by the lines and symbols above the graphs for pairwise comparison (*P < 0.05; **P < 0.01)

The inhibitory effects of FA and Rb1 on platelet surface HMGB1 expression were evaluated. Thrombin stimulation significantly increased HMGB1 expression compared to the blank control. FA potently attenuated this increase with an IC_50_ of 31.03 µM (Fig. 7A, B), whereas Rb1 exhibited a weaker inhibitory effect with an IC_50_ of 508.3 µM (Figure S1B, C). ELISA analysis of HMGB1 in cell supernatants yielded IC_50_ values of 19.28 µM for FA and 175.10 µM for Rb1 in inhibiting HMGB1 release from platelets (Fig. 7C and S1D). These results clearly define the potency and effective concentration window for each compound, with FA being a more potent inhibitor of HMGB1 release. Western blot analysis demonstrated that thrombin activated platelet p38 and ERK1/2, consequently enhancing HMGB1 release from platelets. This activation was attenuated by both FA and GA (Fig. 7D-G). The p38 inhibitor SB203580 and the ERK1/2 inhibitor U0126 effectively suppressed thrombin-induced platelet-derived HMGB1 release. Furthermore, FA was found to counteract the effect of the p38 activator anisomycin on HMGB1 release (Fig. 7H, I). These results indicated that the p38/ERK1/2 pathway played a key role in the FA-mediated inhibition of platelet HMGB1 release. Based on molecular docking and dynamics simulations indicating the stable, high-affinity binding of both FA and Rb1 to PAD4, we assessed their efficacy on PAD4 enzymatic activity in vitro. The IC_50_ values for inhibiting PAD4 activity were 23.01 µM for Rb1 (Fig. 7J) and 101.8 µM for FA (Figure S1E), indicating that Rb1 is a more potent inhibitor of PAD4.

### Synergistic inhibition of NET formation by FA and Rb1

**Fig. 8:**
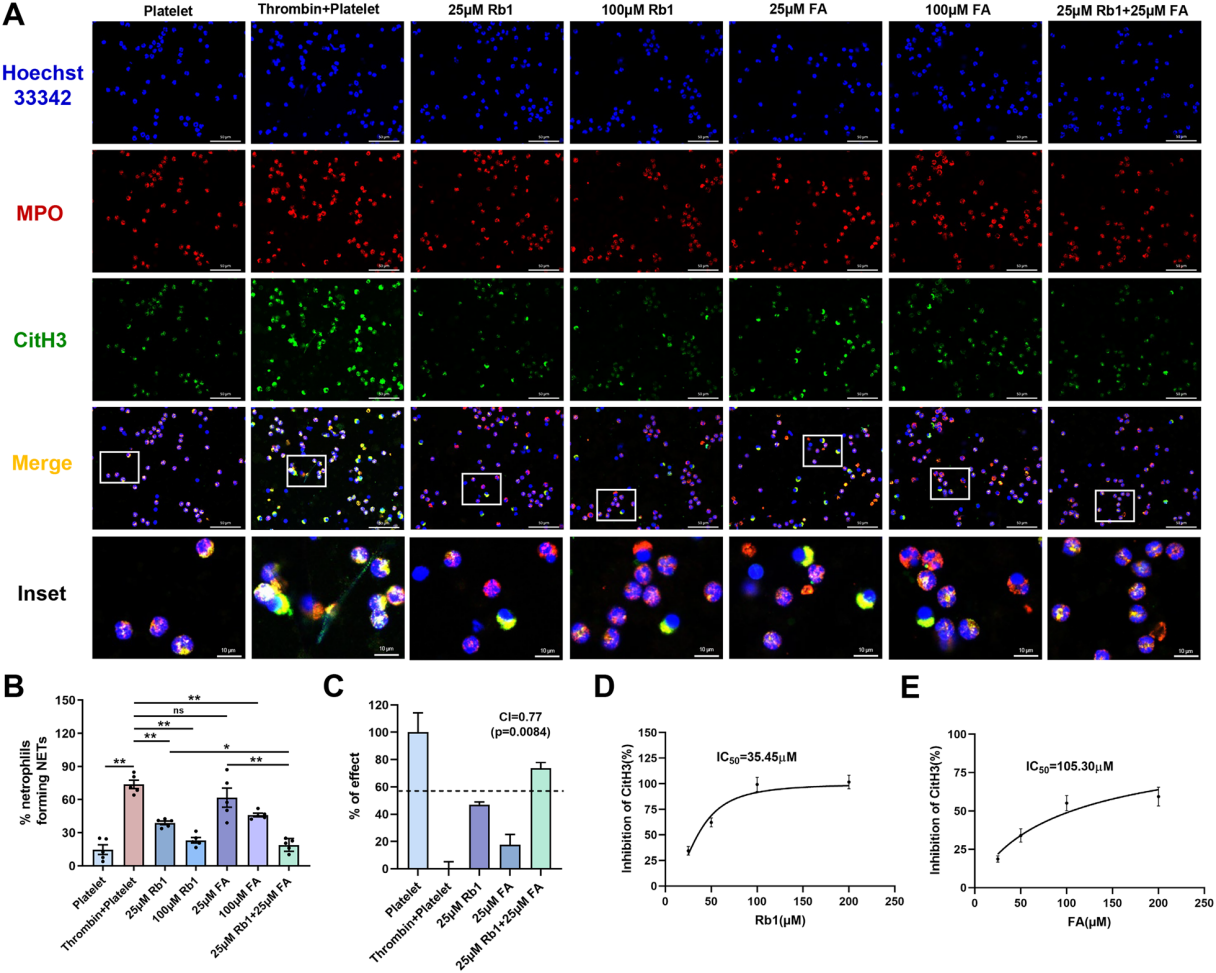
Synergistic inhibition of NET formation by FA and Rb1. **A** Hoechst 33,342 (blue), anti-MPO (red), and anti-CitH3 (green) staining were used to assess NET formation induced by thrombin‐stimulated platelets. Scale bar = 50 μm (Hoechst 33,342, MPO, CitH3 and Merge) or 10 μm (Inset). **B** Quantitative analysis of NET formation (n = 5). **C** Statistical diagram showing the improvement effects on NET formation. The CI was calculated according to Bliss Independence model. The dashed line represents the value of E_FA_ + E_Rb1_—E_FA_ × E_Rb1_. CI = (E_FA_ + E_Rb1_—E_FA_ × E_Rb1_)/ E_FA+Rb1_. A CI value less than 1 indicates the synergistic effects of FA and Rb1. P was measured between E_FA+Rb1_ and E_FA_ + E_Rb1_—E_FA_ × E_Rb1_ by t-test. **D**, **E** Platelets were pretreated for 10 min with either Rb1 or FA. Following this, the platelets were activated for 30 min with thrombin and then incubated for 2 h with neutrophils. After this incubation, NETs were quantified using a CitH3 ELISA. Data are presented as means ± SE. Significance is indicated by the lines and symbols above the graphs for pairwise comparison (*P < 0.05; **P < 0.01; ns, not significant)

Activated platelets induced the NET formation, as characterized by positive staining for MPO and CitH3. Both Rb1 and FA treatment reduced NET formation obviously. At a concentration of 25 µM, Rb1 alone significantly reduced NET formation by approximately 35%. Notably, the combination of 25 µM FA and 25 µM Rb1 exhibited a superior inhibitory effect compared to either agent alone (Fig. 8A, B). A combined index (CI) value of 0.77 was obtained for 25 µM FA and 25 µM Rb1, suggesting high synergistic effects between the two compounds (Fig. 8C). Consistent with these findings, ELISA analysis of CitH3 in cell supernatants yielded IC_50_ values of 35.45 µM for Rb1 and 105.30 µM for FA in inhibiting NET formation (Fig. 8D, E).

### Combination of FA and Rb1 synergistically improves cardiac function and attenuates myocardial NR by inhibiting platelet HMGB1 release and NET formation

**Fig. 9:**
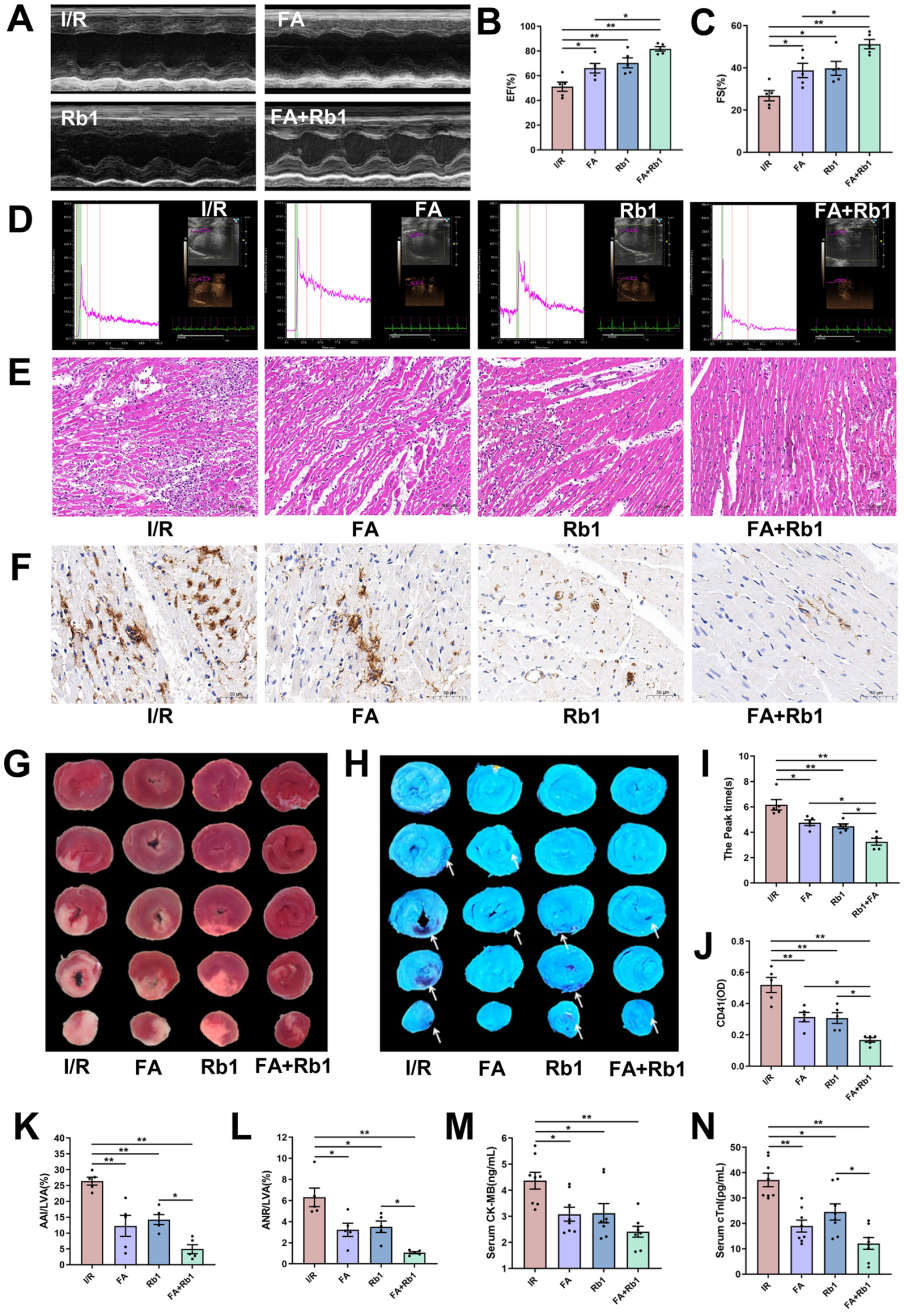
FA in combination with Rb1 alleviate NR. Rats were subjected to 45 min of LAD followed by 24 h of reperfusion or sham surgery. Rats were prophylactically administered with saline, FA (50 mg·kg^−1^ day^−1^), Rb1 (50 mg·kg^−1^ day^−1^), or FA plus Rb1 (both at 50 mg·kg^−1^ day^−1^). **A** Representative images of echocardiography. **B**, **C** Quantitative analysis of left ventricular EF and FS (n = 5). **D** Representative images of real-time MCE. **E** Representative images of HE staining in heart sections (scale bar: 100 µM). **F** Representative images of immunohistochemical staining for CD41 in heart sections (scale bar: 50 µM). **G** TTC staining. **H** Thioflavin S staining. **I** Values of myocardial perfusion peak time (n = 5). **J** Quantitative analysis of CD41 (n = 5). **K** Quantitative analysis of myocardial infarction area (n = 5). **L** Quantitative analysis of NR area (n = 5). **M**, **N** Serum CK-MB and cTnI levels were measured by ELISA (n = 8). Data are presented as means ± SE. Significance is indicated by the lines and symbols above the graphs for pairwise comparison (*P < 0.05; **P < 0.01)

Based on the above results, FA and Rb1 synergistically inhibited NET formation, with FA blocking platelet HMGB1 release via p38/ERK1/2 pathway and Rb1 showing stronger inhibition of PAD4 activity, then their effects on MIRI and NR were assessed in the rat model. Transthoracic echocardiography revealed that compared with the model group, FA, Rb1, or the FA-Rb1 combination exhibited significant improvements in EF and FS. Furthermore, the FA-Rb1 combination led to significantly greater increases in EF and FS compared with FA alone (Fig. 9A-C). Real-time MCE demonstrated a marked reduction in the peak time of myocardial perfusion in all treatment groups relative to untreated model rats. Importantly, the FA-Rb1 combination shortened the peak perfusion time compared with either agent alone (Fig. 9D, I). The CI value of the peak time demonstrated that FA and Rb1 exerted synergistic effects in improving myocardial microcirculation (Figure S2A). Histological examination indicated that inflammatory cell infiltration was suppressed in all treatment groups (Fig. 9E). Immunohistochemical staining revealed a significant decrease in microthrombus formation under treatment, with the combination of FA and Rb1 showing a more substantial reduction than either agent alone (Fig. 9F, J). CI value of CD41 expression indicated the synergistic effects of FA and Rb1 on microthrombosis (Figure S2B). As shown by TTC staining, both individual treatments reduced the myocardial infarct area after I/R injury, and FA-Rb1 combination was more effective than Rb1 alone (Fig. 9G, K). Similarly, Thioflavin S staining results demonstrated a marked reduction in the NR area in all treatment groups. The FA-Rb1 combination achieved the best effect, reducing the NR area by 83.2 ± 14.9%, exceeding the efficacy of either FA (48.8 ± 16.2%) or Rb1 (44.2 ± 16.2%) (Fig. 9H, L). ELISA further confirmed that FA, Rb1, and their combination significantly reduced CK-MB and cTnI levels, with the  combination treatment leading to a more pronounced reduction in cTnI levels compared with Rb1 alone (Fig. 9M, N).

**Fig. 10:**
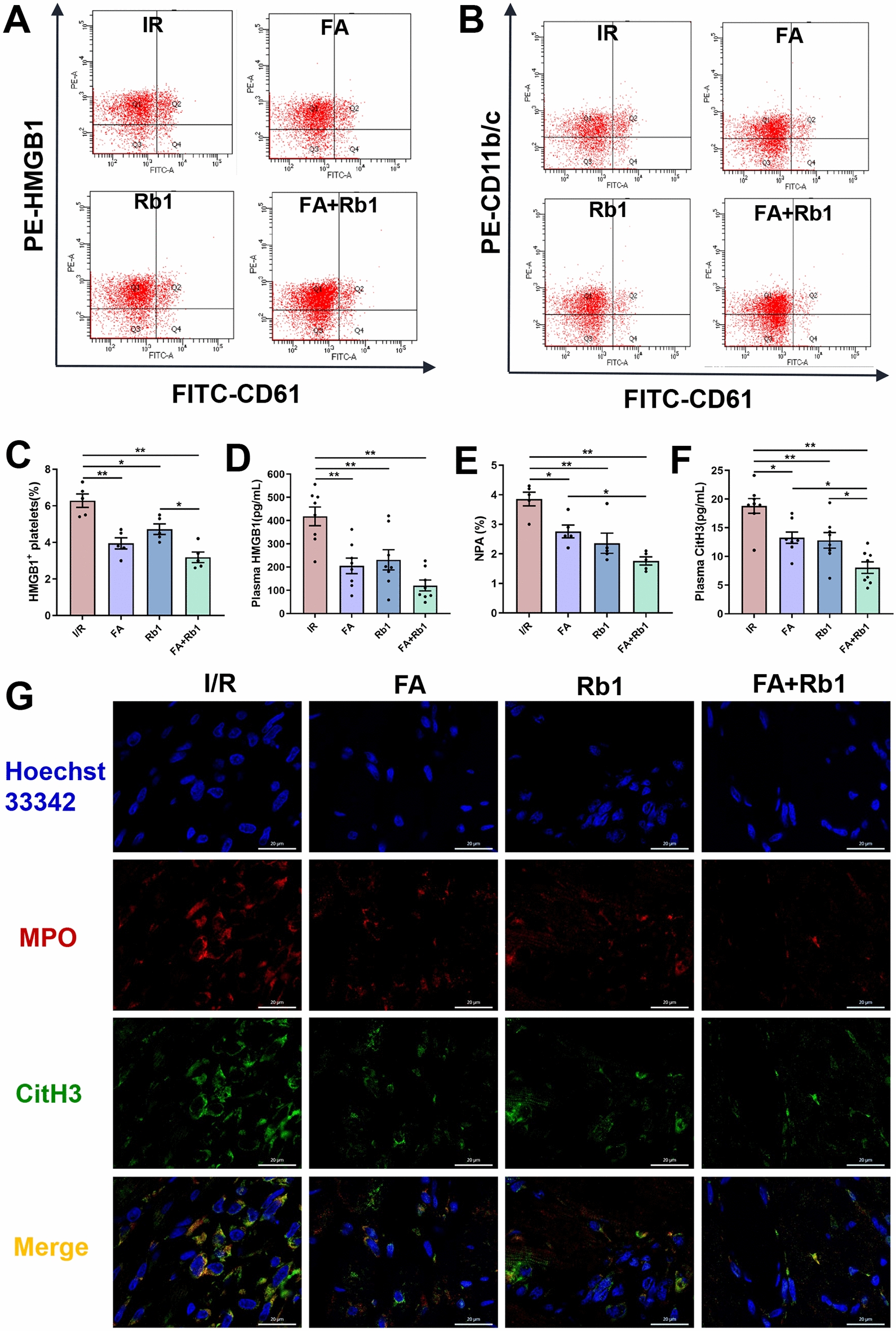
FA in combination with Rb1 alleviated NR in rats by inhibiting platelet HMGB1 release and NET formation. Rats were subjected to 45 min of LAD followed by 24 h of reperfusion or sham surgery. Rats were prophylactically administered with saline, FA (50 mg·kg^−1^ day^−1^), Rb1 (50 mg·kg^−1^ day^−1^), or FA plus Rb1 (both at 50 mg·kg^−1^ day^−1^). **A**, **B** Representative flow cytometry scatter plot of platelet HMGB1 expression and NPA levels in the diluted whole blood. **C** Quantitative analysis of platelet surface HMGB1. **D** Plasma HMGB1 levels was measured by ELISA (n = 8). **E** Quantitative analysis of NPA levels (n = 5). **F** Plasma CitH3 levels was measured by ELISA (n = 8). **G** Representative immunofluorescence images of NET formation stained with MPO (red), CitH3 (green), and Hoechst 33,342 (blue) (scale bar: 20 μm). Data are presented as means ± SE. Significance is indicated by the lines and symbols above the graphs for pairwise comparison (*P < 0.05; **P < 0.01)

Flow cytometric analysis showed that HMGB1 expression on the platelet surface was significantly elevated in the model group. This increase was markedly suppressed in all treatment groups, with the FA-Rb1 combination being more effective than Rb1 alone (Fig. 10A, C). ELISA further confirmed that FA, Rb1, and their combination significantly reduced HMGB1 levels in plasma (Fig. 10D). All three treatments effectively reduced the circulating NPA proportion, with the FA-Rb1 combination inducing a significantly greater reduction than FA alone (Fig. 10B, E). Immunofluorescence staining of myocardial tissue for MPO and CitH3 revealed that all treatments significantly inhibited NET formation compared with the model group (Fig. 10G). FA and Rb1 significantly reduced plasma CitH3 levels by 29.5 ± 7.1% and 32.0 ± 7.4%, respectively; and the FA-Rb1 combination exhibited a more pronounced protective effect, with a reduction of 57.2 ± 6.5% (Fig. 10F). A CI value of 0**.**97 was obtained for FA and Rb1, indicating synergistic effects between the two compounds (Figure S3C). While previous studies demonstrating the dose-dependent cardioprotective effects of Rb1 and FA individually [39, 52], this study shows for the first time that their co-administration exerts a synergistic effect against NR phenomena.

## Discussion

The NR phenomenon remains a significant clinical challenge that limits the benefits of reperfusion therapy in AMI patients. NR occurs following sustained occlusion and subsequent reperfusion. Previous studies have simulated the reperfusion and NR phenomenon by surgically ligating the LAD coronary artery followed by the release of the ligation in rats model [53, 54]. Considering that the standard ischemic period for LAD ligation ranges from 30 to 60 min and that neutrophil infiltration peaks within 24 h of reperfusion [55], we employed a rat I/R model comprising 45 min of ischemia followed by 24 h of reperfusion. Myocardial echocardiography, HE staining, and infarct area analysis indicated that I/R injury led significant myocardial damage. To specifically evaluate the NR phenomenon, we utilized real-time MCE, a technique clinically validated for the noninvasive evaluation of myocardial capillary perfusion [56]. The results from both real-time MCE and thioflavin S staining consistently and conclusively demonstrated the occurrence of the NR phenomenon in the myocardial I/R model rats.

Despite being anucleate, platelets express and store HMGB1, which is translocated to the cell surface and released into the extracellular space following platelet activation [21]. In the extracellular milieu, HMGB1 acts as a potent damage-associated molecular pattern molecule that activates immune cells, stimulates the production of pro-inflammatory mediators [57], and enhances coagulation pathways [58] during tissue injury. Consistent with this mechanism, the present data evidenced that myocardial I/R injury triggers HMGB1 release and platelet HMGB1 expression. Activated platelets form aggregates with neutrophils within the microvasculature, a process that amplifies inflammatory responses, promotes inflammatory cell infiltration, and induces immune-mediated cardiomyocyte death [59]. NPAs are a common feature in occluded vessels in AMI and other injury models [60, 61]. Flow cytometric analysis in this study revealed a significant increase in circulating NPA levels in I/R model rats compared with sham controls, and consistent with the recent findings [62]. Concurrently, extensive NET formation was observed in the ischemic myocardium, together with the elevated serum CitH3 levels, contributing to microvascular plugging. Importantly, HMGB1 and NETs have been shown to co-localize in coronary thrombi of AMI patients [14], providing a direct link between inflammatory and thrombotic processes. The causal involvement of HMGB1 and NETs in the NR phenomenon was demonstrated in our study through interventional experiments using the HMGB1 inhibitor GA and DNase I, a NET-degrading enzyme. Both interventions significantly attenuated cardiac dysfunction, improved microvascular perfusion, reduced infarct and NR area, underscoring HMGB1 and NETs as valid therapeutic targets for mitigating NR.

Immune cell infiltration analysis of a GEO dataset revealed that neutrophil infiltration levels were considerably higher in the peripheral blood of ACS patients than that of healthy adults, with key genes such as PAD4 showing a strong correlation with neutrophil levels. Network pharmacology identifed both PAD4 and HMGB1 as core targets within the PPI network of the FA and Rb1 combination. The functional enrichment analyses strongly suggest that the therapeutic potential of FA and Rb1 against ACS involves the suppression of NET formation and several inflammatory signaling pathways. The computational validation of this mechanism through molecular docking and dynamics simulations strengthens the prediction. The high binding affinities and stable complex formation of FA and Rb1 with both HMGB1 and PAD4, as evidenced by favorable free energies, low RMSD/RMSF fluctuations, and persistent hydrogen bonding, provide a structural basis for their inhibitory action. By potentially blocking HMGB1 pro-inflammatory signaling and inhibiting PAD4 during NET formation, the FA-Rb1 combination thus is deemed as a promising multi-target strategy.

A key finding of this study is the complementary pharmacological profiles of FA and Rb1. FA demonstrated as a potent inhibitor of thrombin-induced HMGB1 release from platelets, with an IC_50_ of 19.28 µM. Based on evidence that thrombin activates platelets and subsequent HMGB1 release via the p38/ERK1/2 pathway [63, 64], we found that FA (20 µM) potently inhibited thrombin-induced phosphorylation of p38 and ERK1/2, thereby reducing HMGB1 release. This mechanism was validated using the p38 inhibitor SB203580 and the ERK1/2 inhibitor U0126, which mimicked FA effect, and FA was found to antagonize the effect of anisomycin, a p38 activator, on the thrombin induced platelet HMGB1 release, indicating that FA acts through suppression of the p38/ERK1/2 pathway to reduce HMGB1 release. In contrast, Rb1 functioned as a potent inhibitor of PAD4 enzymatic activity with an IC_50_ of 23.01 µM, which is essential for chromatin decondensation in NETosis. Moreover, the combination of 25 µM FA and 25 µM Rb1 exhibited a synergistic inhibitory effect, leading to a greater suppression than either agent alone. The FA-Rb1 combination demonstrated significantly enhanced therapeutic efficacy in vivo, exerted improved cardiac function (EF, FS) and myocardial perfusion more effectively than FA alone, and more pronounced reduction in myocardial infarct size, the NR area, and plasma cTnI levels than Rb1 alone. Notably, the FA-Rb1 combination reduced the NR area by 83.2 ± 14.9%, exceeding the efficacy of the positive controls GA (69.6 ± 15.2%) and Dnase I (78.8 ± 15.0%). we demonstrated for the first time that the combination of FA and Rb1 significantly alleviated the NR phenomenon and reduced infarct size in a rat model of MIRI. The underlying mechanism involved the synergistic inhibition of two interactive pathways, the release of HMGB1 from activated platelets and the subsequent NET formation. This combination strategy, hinted by a classic herbal pair in TCM, presents a novel and promising therapeutic approach for mitigating microvascular obstruction in myocardial NR.

This study demonstrated that the combination of FA and Rb1 effectively ameliorated myocardial NR, yet several limitations should be addressed. First, future investigations utilizing platelet-specific HMGB1 transgenic mouse models could yield more unequivocal insights into the mechanisms driving NET formation. Furthermore, although the inhibition of NET formation emerged as a central pathway, the involvement of additional mechanisms in the improvement of myocardial NR remains possible and requires further elucidation. Last, comprehensive pharmacokinetic studies on the FA-Rb1 combination are necessary to provide a foundation for its therapeutic development. This study also encourages a reflective examination of the advantages and limitations of multi-compound pharmacological strategies. Combination therapies offer a promising direction for addressing multifactorial diseases, reducing the risk of compensatory mechanisms, or achieving enhanced efficacy compared with monotherapy [65, 66]. Additionally, combination therapeutic benefits may be achieved with reduced adverse effects through the use of submaximal drug dosages [67]. However, challenges remain in deciphering individual contributions of each compound and translational hurdles in extrapolating findings from preclinical models to clinical settings.

## Conclusion

The combination of FA and Rb1 demonstrated an enhanced cardioprotective effect, significantly reducing myocardial infarct size and the NR area in a rat model of MIRI. Our findings revealed complementary mechanisms, that is, FA attenuated microthrombi by suppressing platelet HMGB1 release via p38/ERK1/2 signaling, while Rb1 abrogated NETosis by inhibiting PAD4. This combination strategy, hinted by a classic herbal pair in TCM, presents a novel and promising therapeutic approach for mitigating microvascular obstruction in myocardial NR.

## Supplementary Information

Supplementary file 1.

Supplementary file 2.

## Data Availability

All the data for this study are available from the corresponding author upon rational request.

## Abbreviations

FA: Ferulic acid
Rb1: Ginsenoside Rb1
NR: No-reflow
AMI: Acute myocardial infarction
NETs: Neutrophil extracellular traps
HMGB1: High mobility group box-1
PAD4: Protein arginine deiminase 4
MIRI: Myocardial ischemia/reperfusion injury
GA: Glycyrrhizic acid
TCM: Traditional Chinese Medicine
MCE: Myocardial contrast echocardiography
CK-MB: Creatine kinase-MB
cTnI: Cardiac troponin I
TTC: 2,3,5-Triphenyltetrazolium chloride
LAD: Left anterior descending
EF: Ejection fraction
FS: Fractional shortening
HE: Hematoxylin and eosin
CI: Combination index
ACS: Acute coronary syndrome
GEO: Gene expression omnibus
DEGs: Differentially expressed genes
GO: Gene ontology
KEGG: Kyoto encyclopedia of genes and genomes
DEGs: Differentially expressed genes
PPI: Protein–protein interaction
MD: Molecular dynamics
RMSD: Root mean square deviation
RMSF: Root mean square fluctuation
SASA: Solvent accessible surface area
RG: Radius of gyration
HB: Hydrogen bonds
ACD: Acid citrate dextrose
EDTA: Ethylenediaminetetraacetic acid
NPA: Neutrophil-platelet aggregates
