# Supplementary material for: Ferulic acid in combination with ginsenoside Rb1 alleviates myocardial no-reflow by inhibiting platelet HMGB1 release and NET formation
Source: Chin Med. 2026 Jan 8;21:16. doi: 10.1186/s13020-025-01303-x (PMC12781747; doi:10.1186/s13020-025-01303-x)
Supplement: Supplementary file 1 [file 13020_2025_1303_MOESM1_ESM.docx]

**
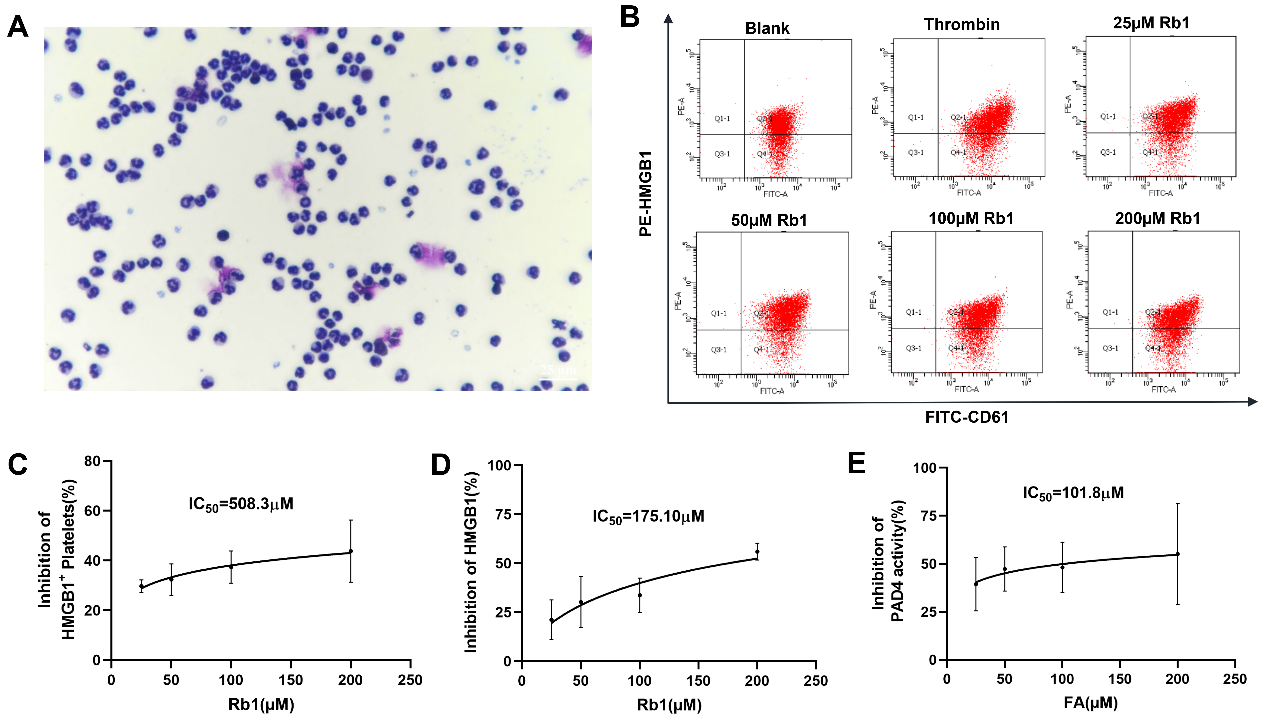
**

**Figure S1.** (A) Neutrophil purity was established to be routinely >90% as determined by wright-giemsa staining (scale bar: 25 μm). (B) Representative flow cytometry scatter plot of platelet HMGB1 expression on washed platelets isolated from rats. (C) The effect of Rb1 on thrombin-induced HMGB1 expression on the surface of platelets. (D) HMGB1 levels in conditioned media derived from resting and activated platelets were also measured by ELISA (n = 5). (E) Enzymatic activity of PAD4 was measured in a cell-free assay upon co-incubation of FA (25, 50, 100, and 200 μM) with the PAD4-substrate (n=5). Data are presented as means±SE.


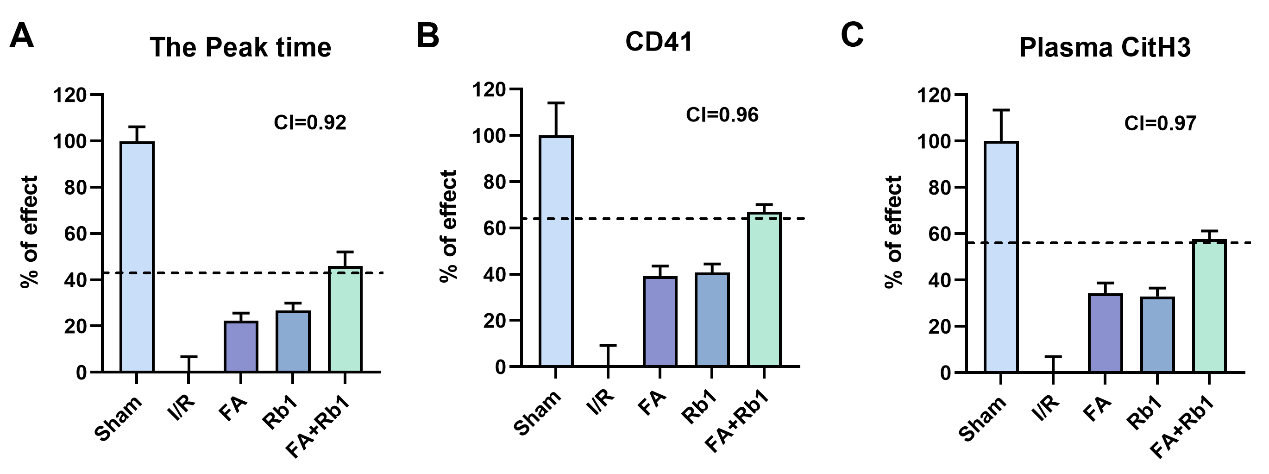


**Figure S2.** (A-C) Statistical diagram showing the improvement effects on the peak perfusion time, microthrombus and NET formation. The combination index was calculated according to Bliss Independence model. The dashed line represents the value of E_FA_+E_Rb1_ - E_FA_× E_Rb1_. CI= (E_FA_+E_Rb1_ - E_FA_× E_Rb1_)/ E_FA+Rb1_. A CI value less than 1 indicates the synergistic effects of FA and Rb1.
