# Supplementary material for: Ferulic acid in combination with ginsenoside Rb1 alleviates myocardial no-reflow by inhibiting platelet HMGB1 release and NET formation
Source: Chin Med. 2026 Jan 8;21:16. doi: 10.1186/s13020-025-01303-x (PMC12781747; doi:10.1186/s13020-025-01303-x)

# Marker used: Thermo Scientific 26616

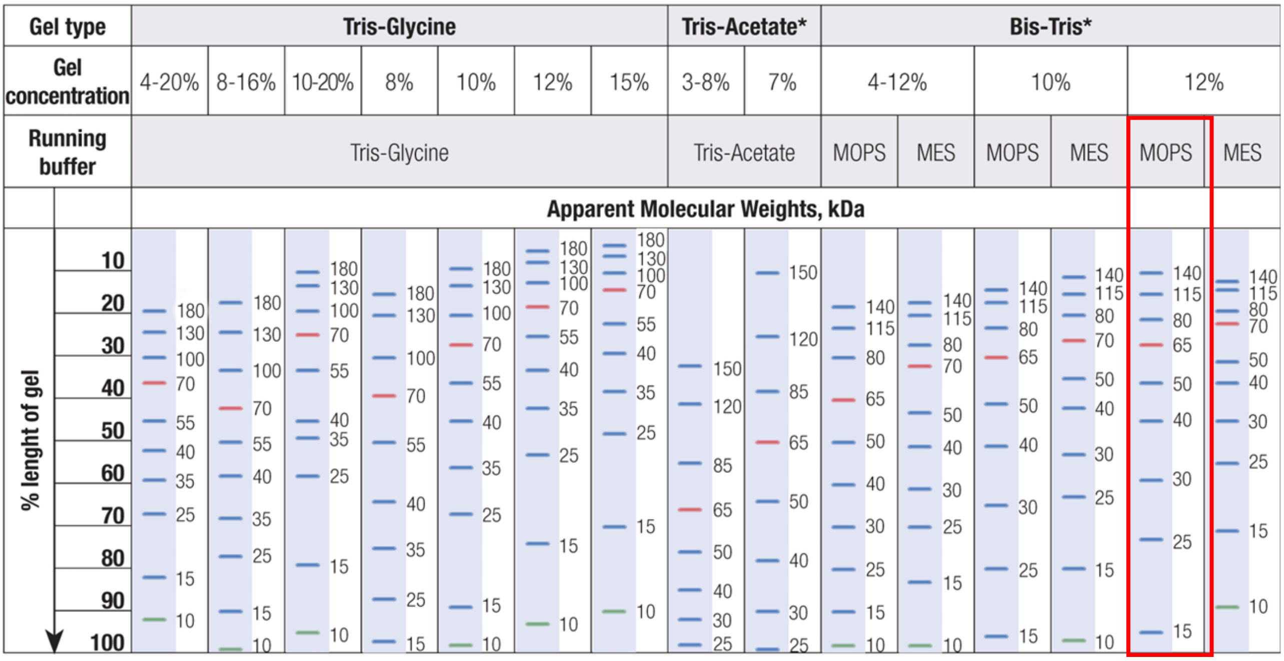


* migration patterns were determined using respective NuPAGE® precast gels.

## WB images for Figure 8D (p-p38) Replicate 1

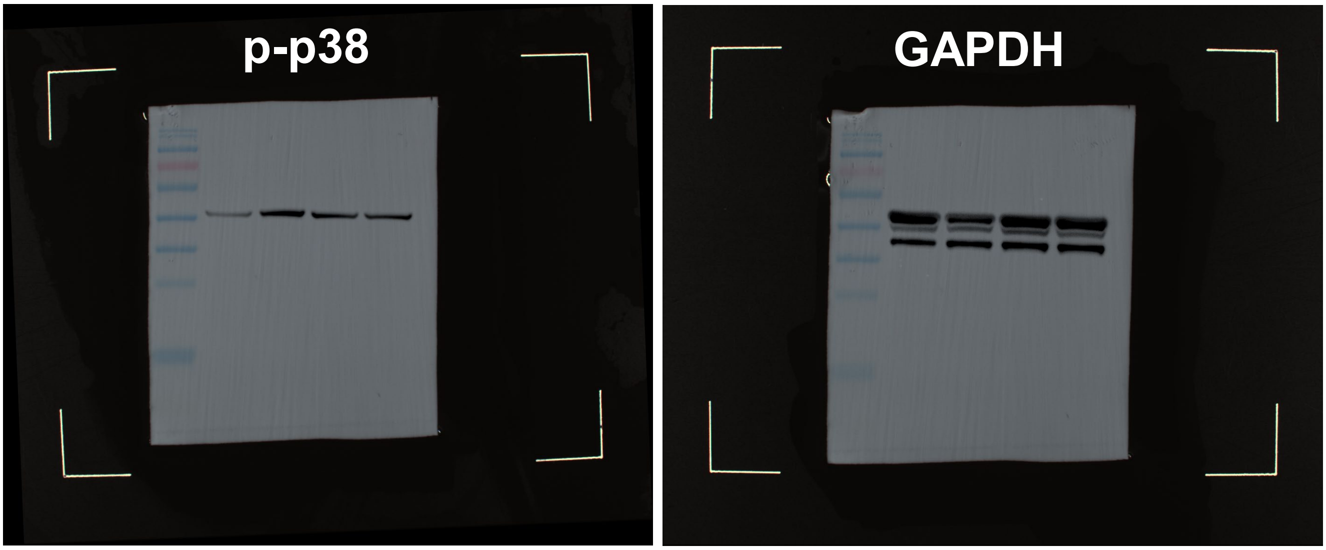


## WB images for Figure 8D (p-p38) Replicate 2

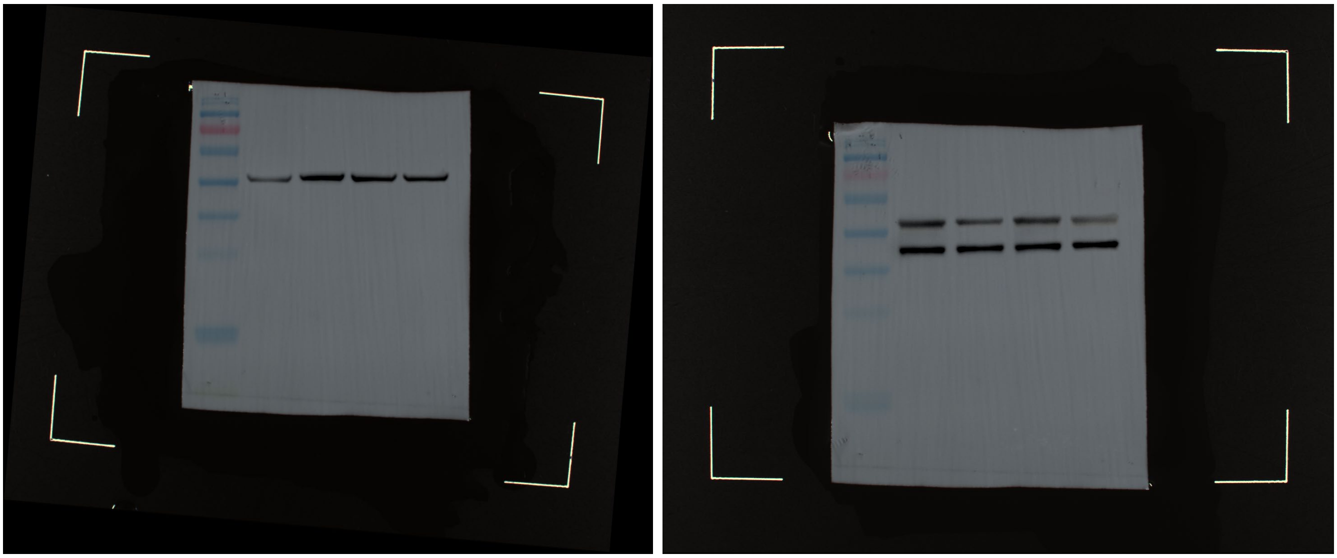

## WB images for Figure 8D (p-p38) Replicate 3

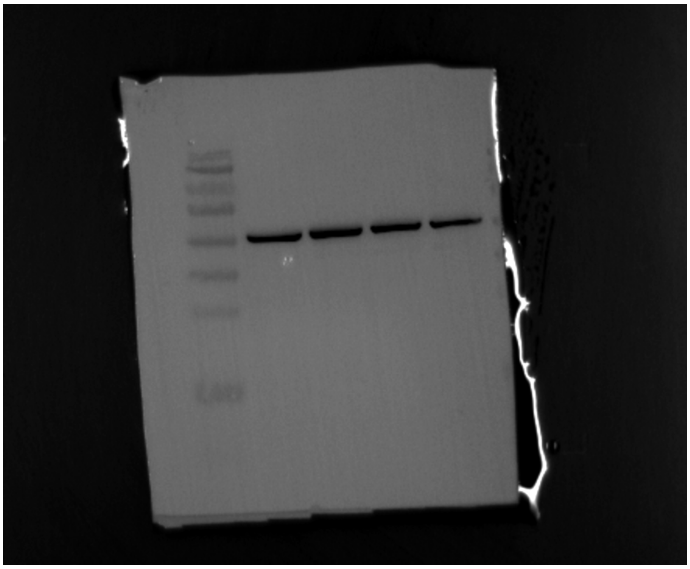
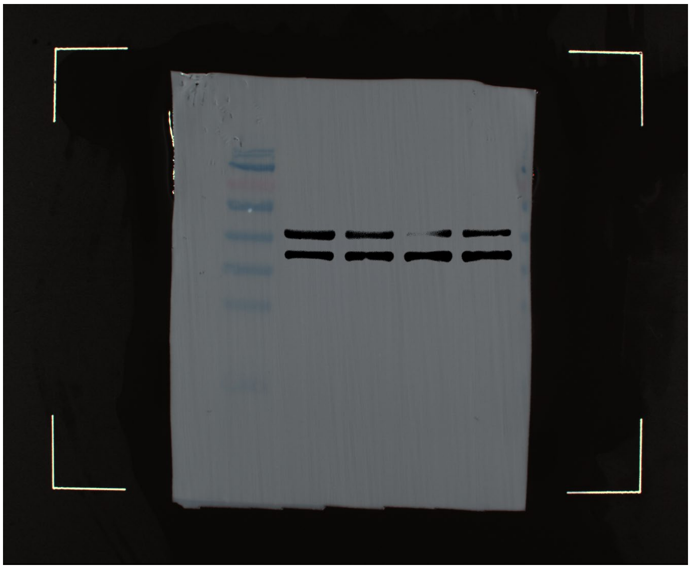

## WB images for Figure 8D (p-p38) Replicate 4

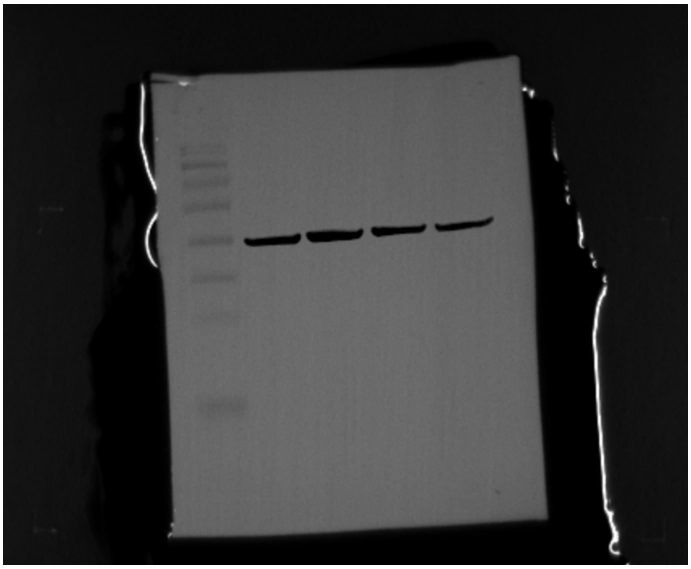
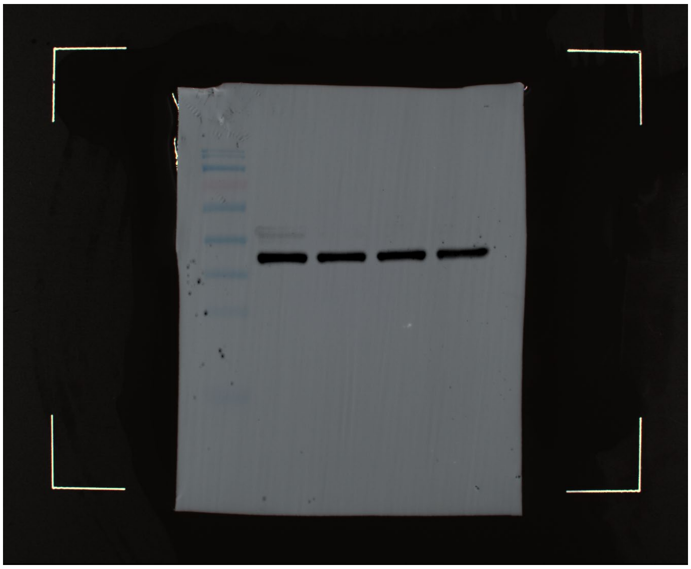

## WB images for Figure 8D (p-p38) Replicate 5

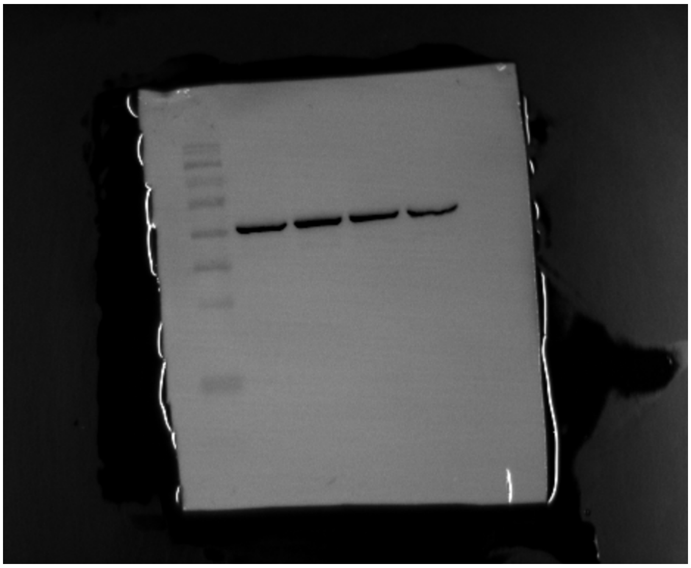
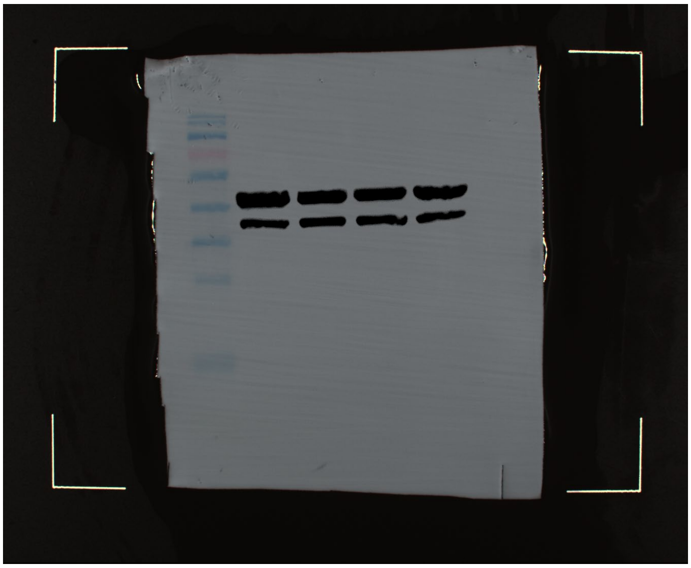

WB images for Figure 8D (p38) Replicate 1

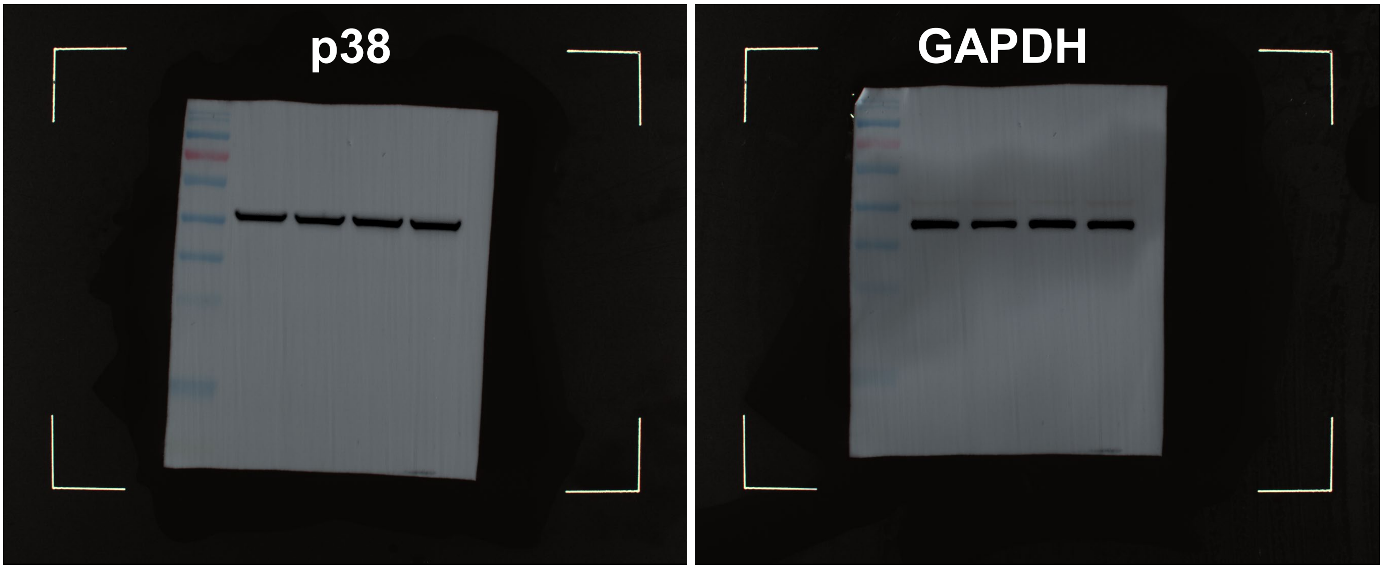


WB images for Figure 8D (p38) Replicate 2

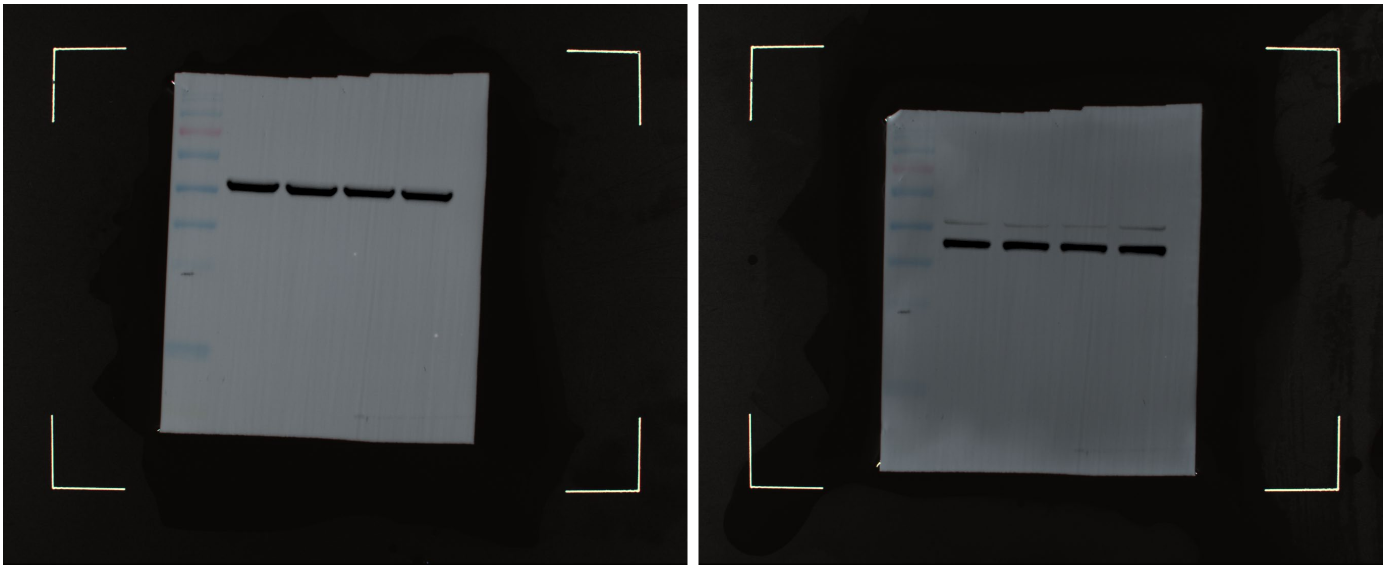

WB images for Figure 8D (p38) Replicate 3

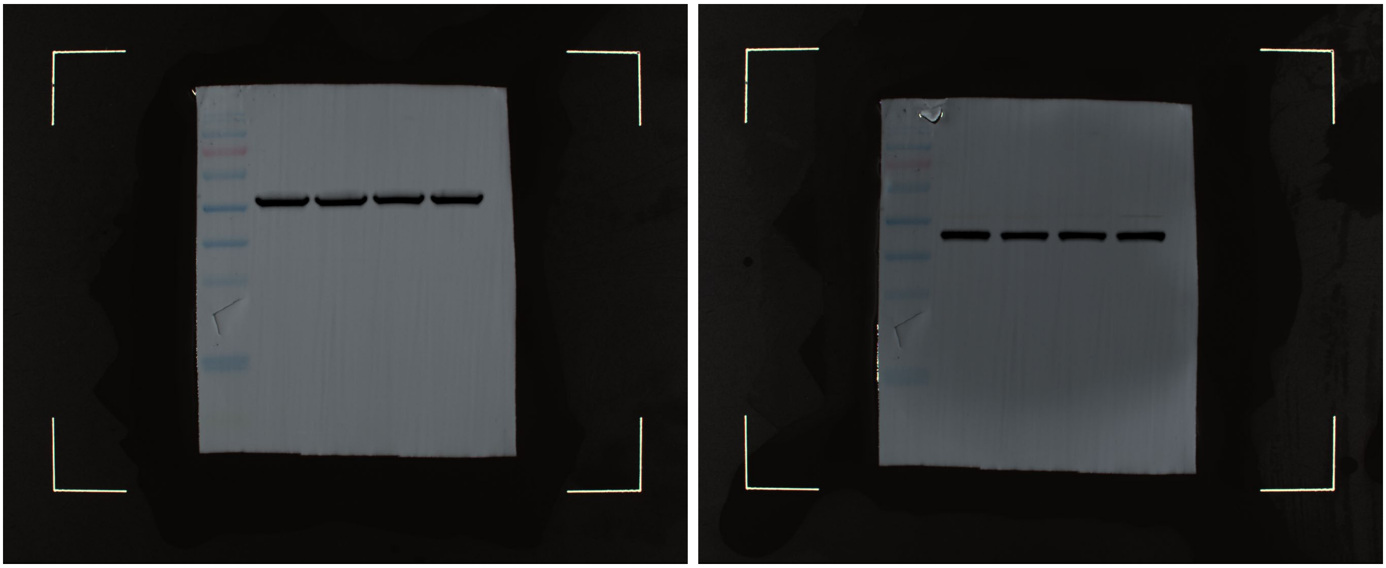

## WB images for Figure 8D (p38) Replicate 4

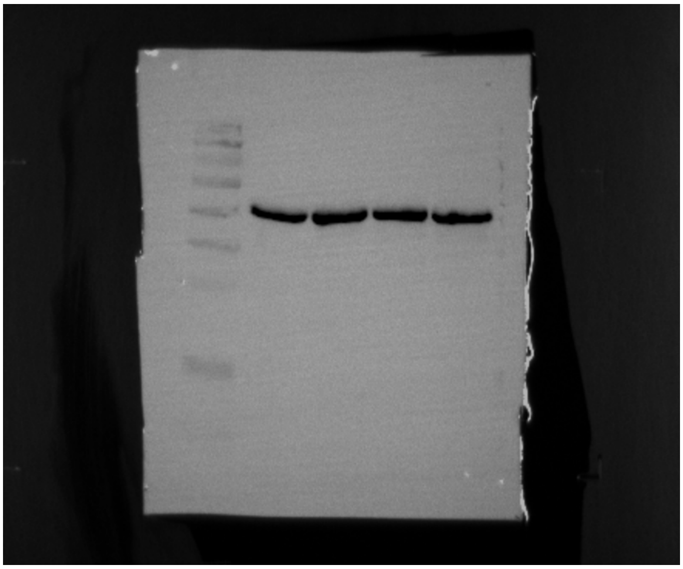

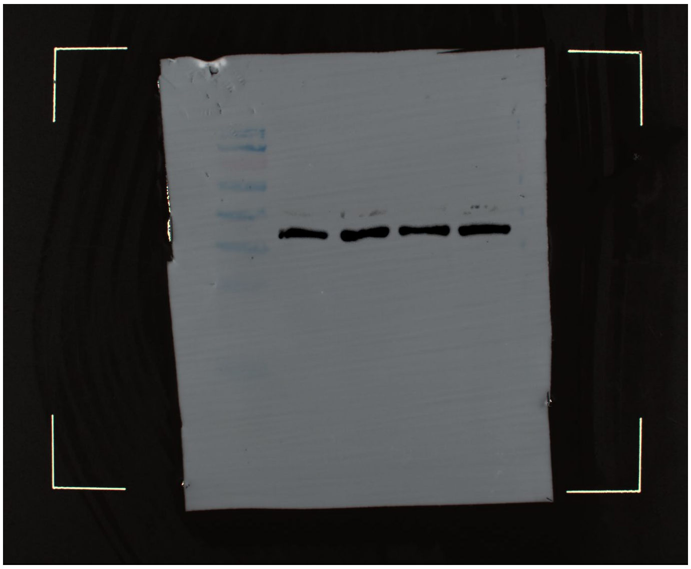

## WB images for Figure 8D (p38) Replicate 5

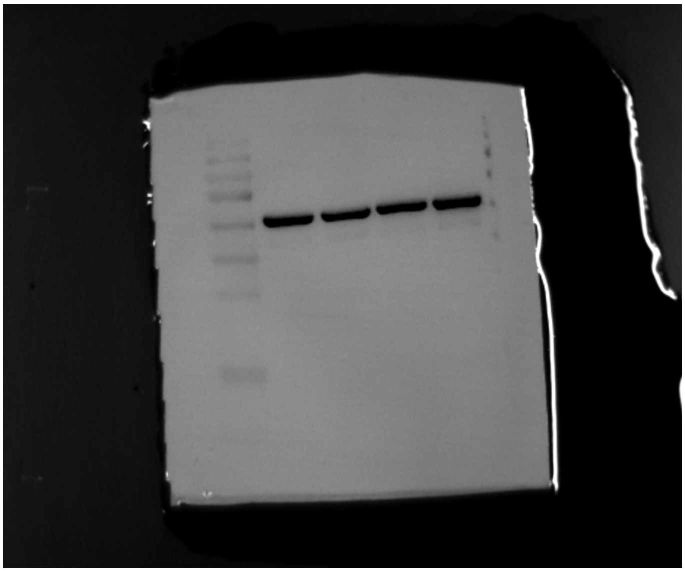

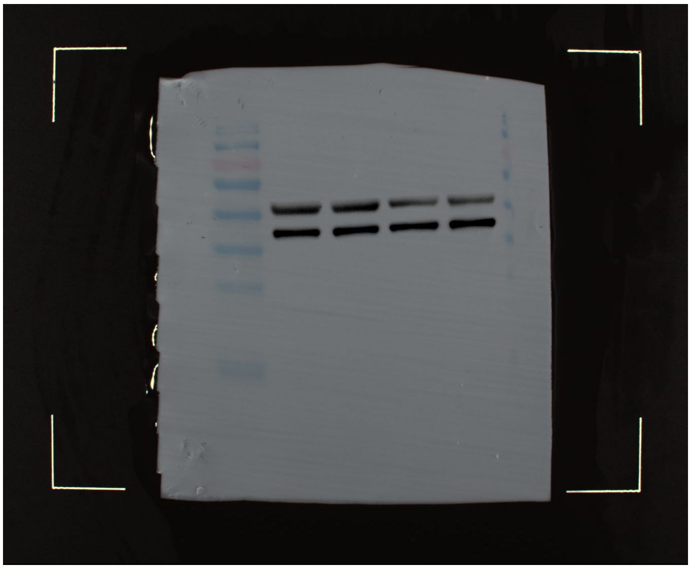

## WB images for Figure 8D (p-ERK1/2) Replicate 1

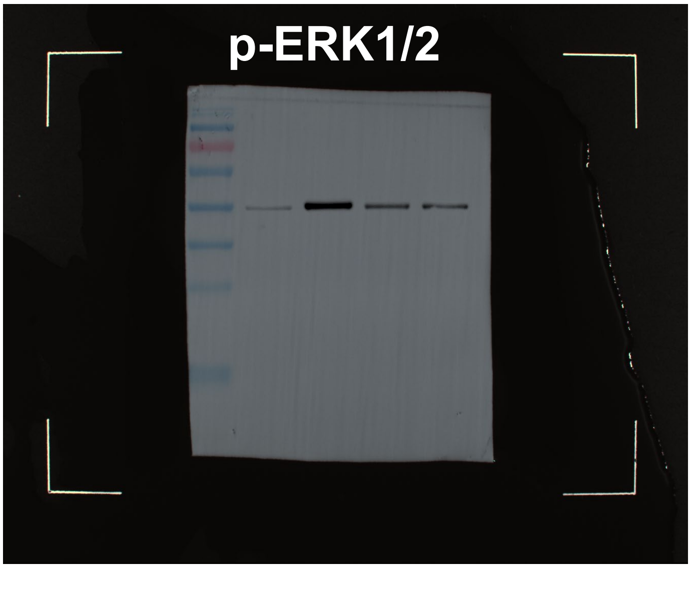


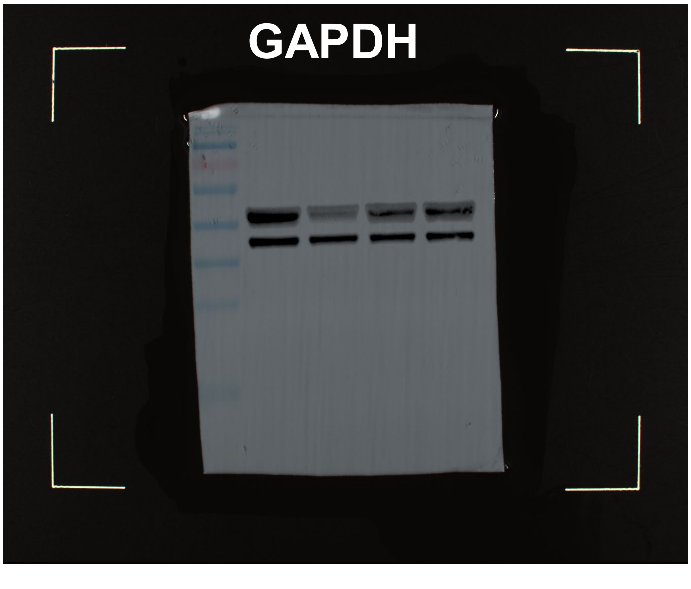

WB images for Figure 8D (p-ERK1/2) Replicate 2

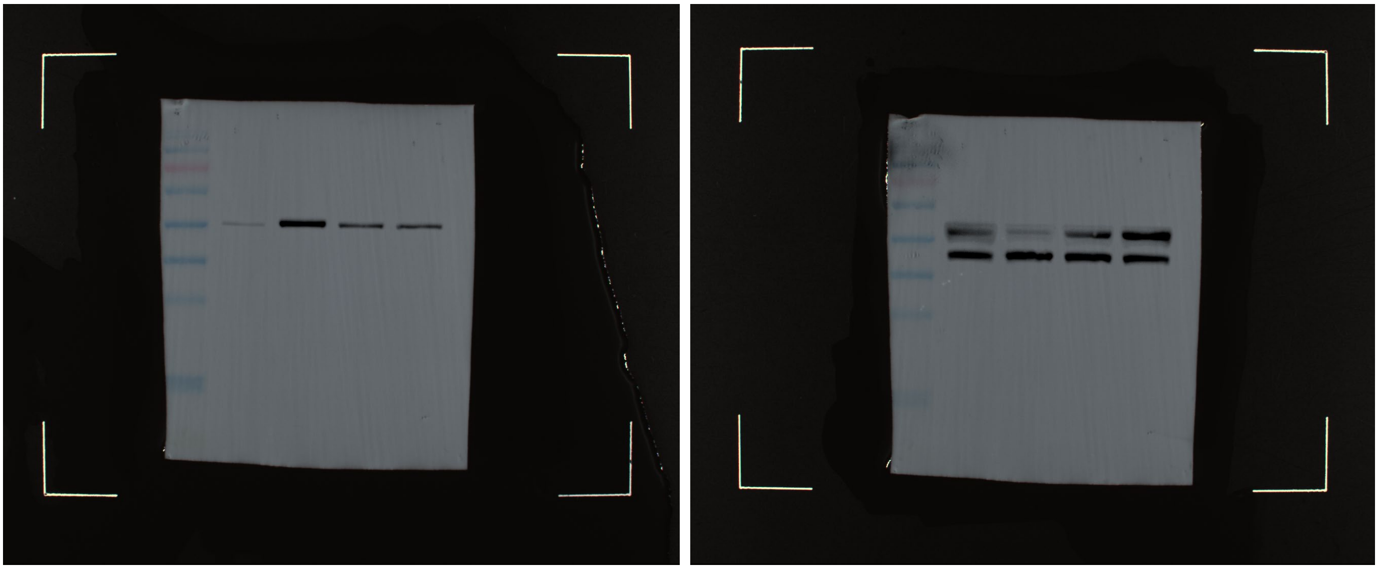

WB images for Figure 8D (p-ERK1/2) Replicate 3

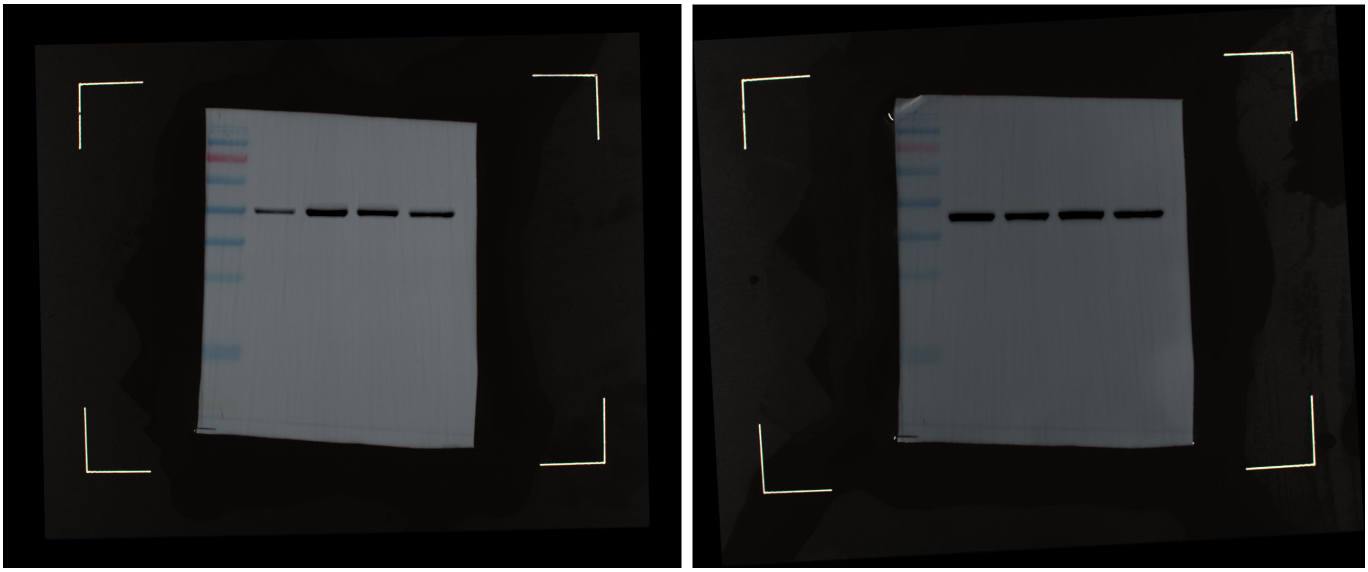

WB images for Figure 8D (p-ERK1/2) Replicate 4

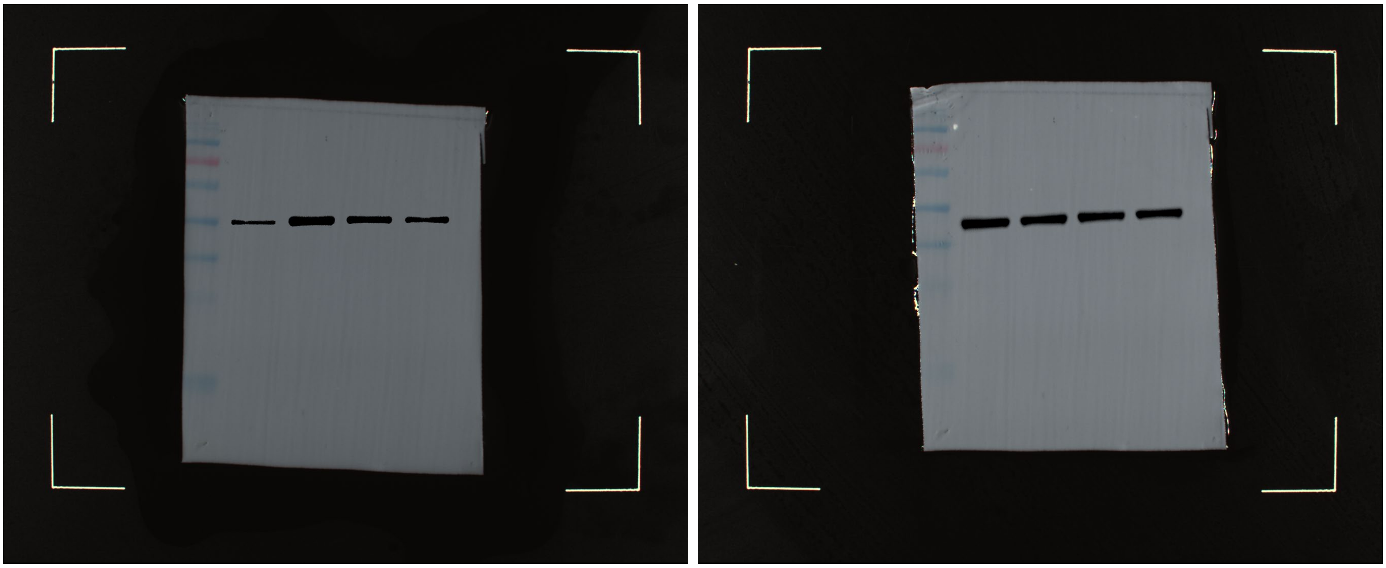

WB images for Figure 8D (p-ERK1/2) Replicate 5

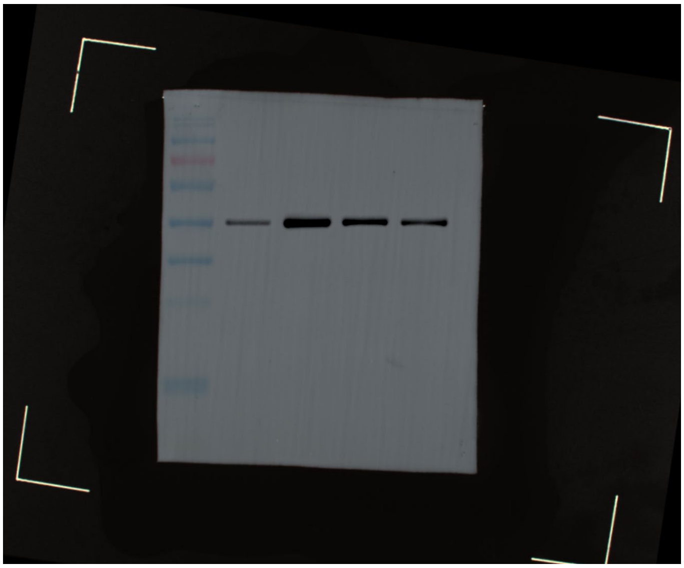

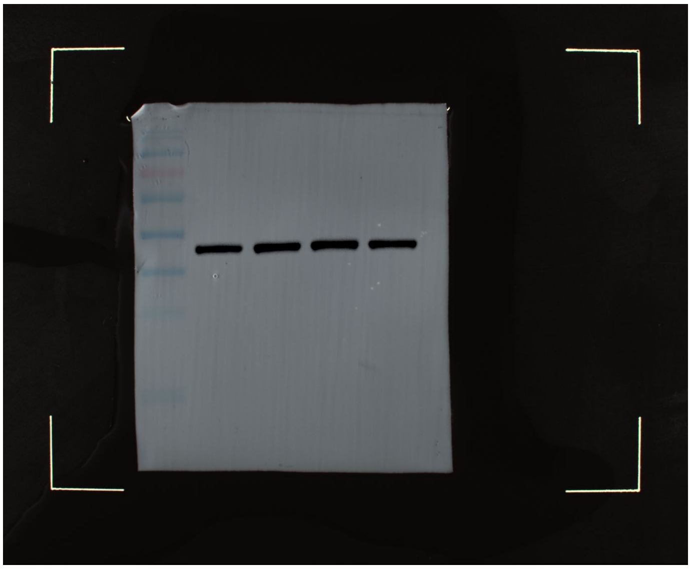

WB images for Figure 8D (ERK1/2) Replicate 1

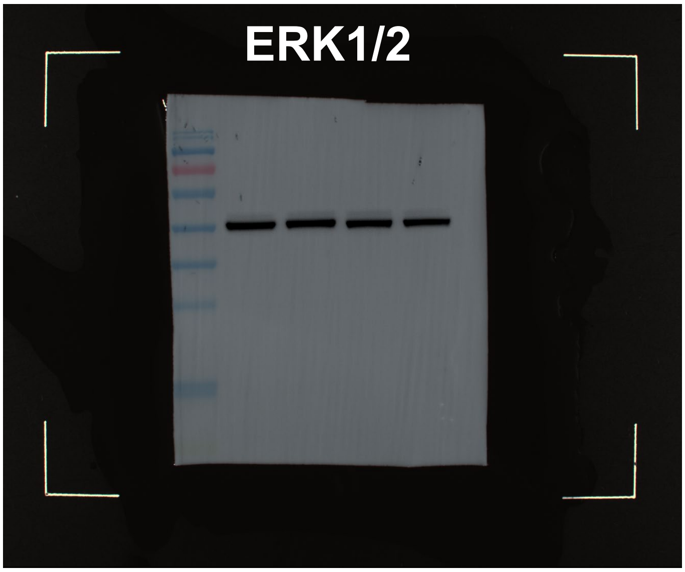


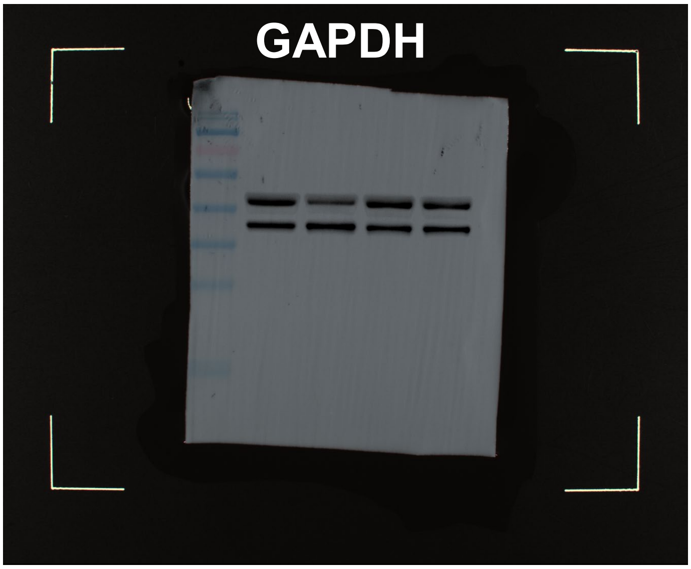


WB images for Figure 8D (ERK1/2) Replicate 2

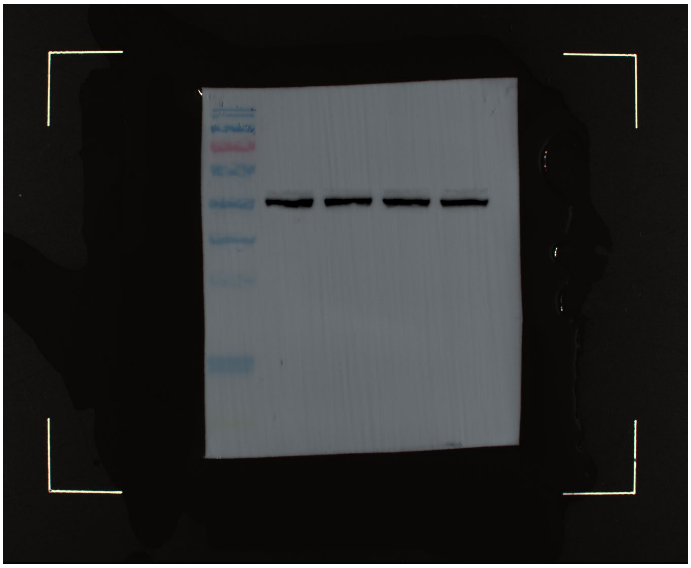

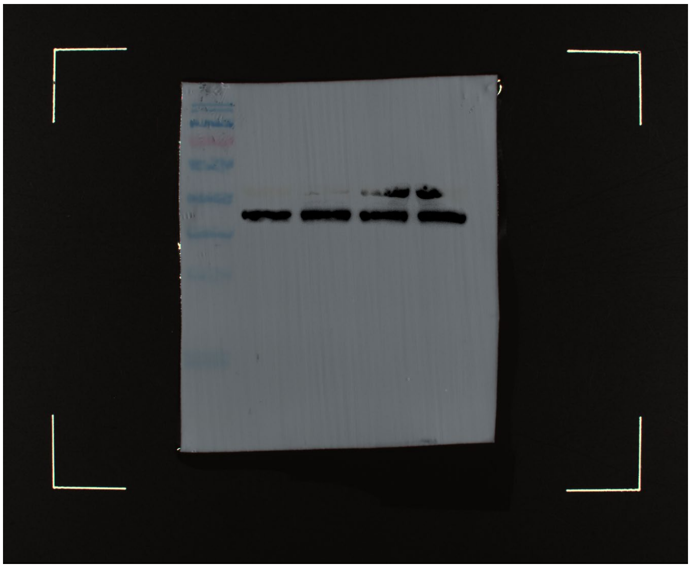

WB images for Figure 8D (ERK1/2) Replicate 3

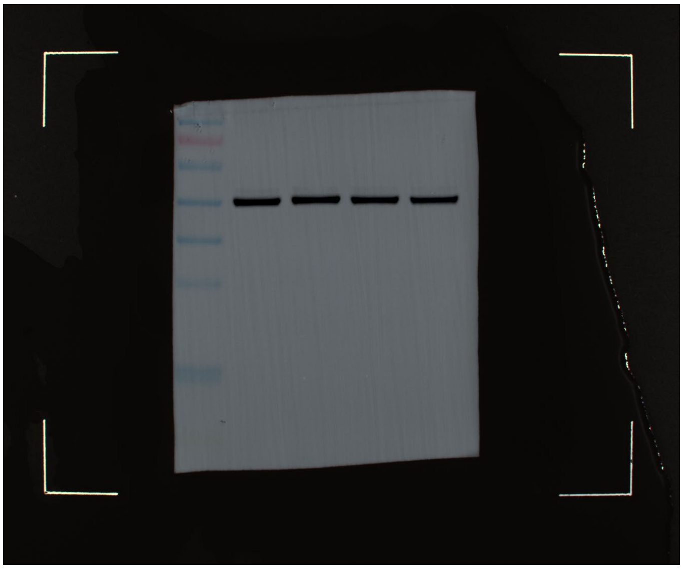

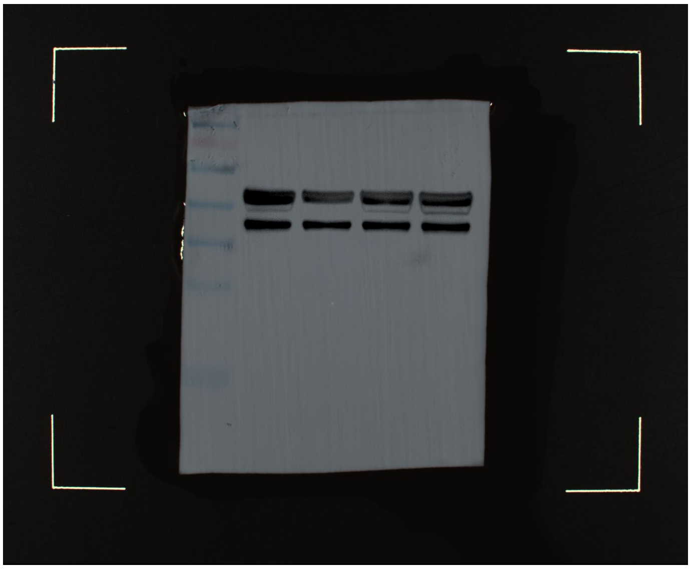

WB images for Figure 8D (ERK1/2) Replicate 4

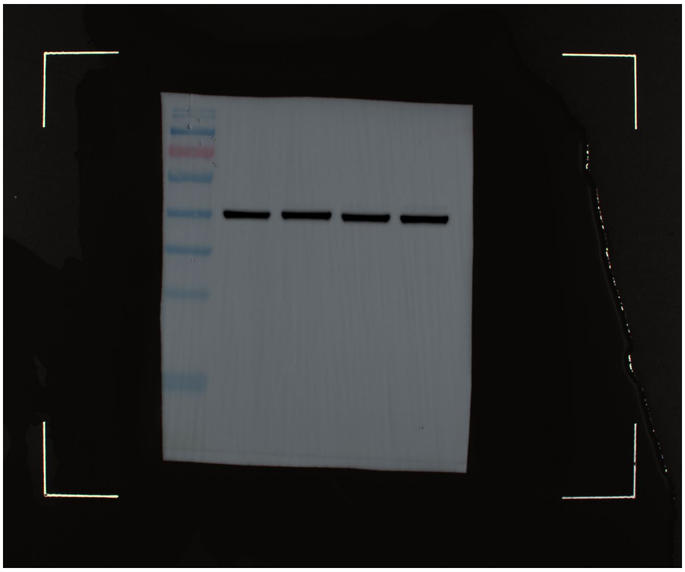

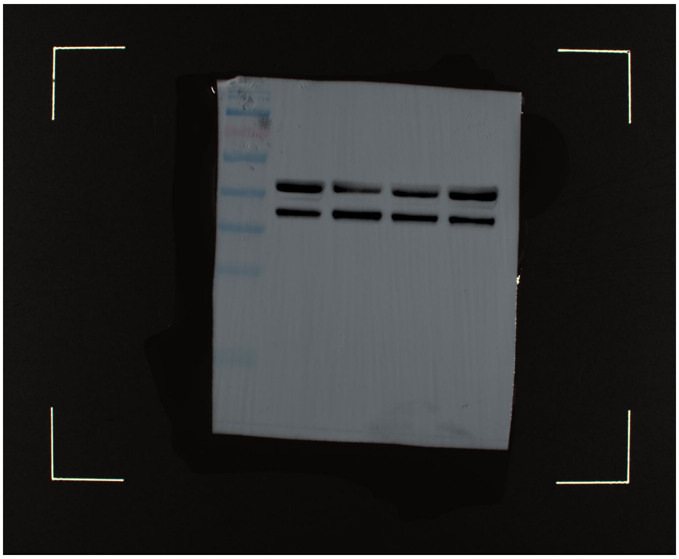

WB images for Figure 8D (ERK1/2) Replicate 5

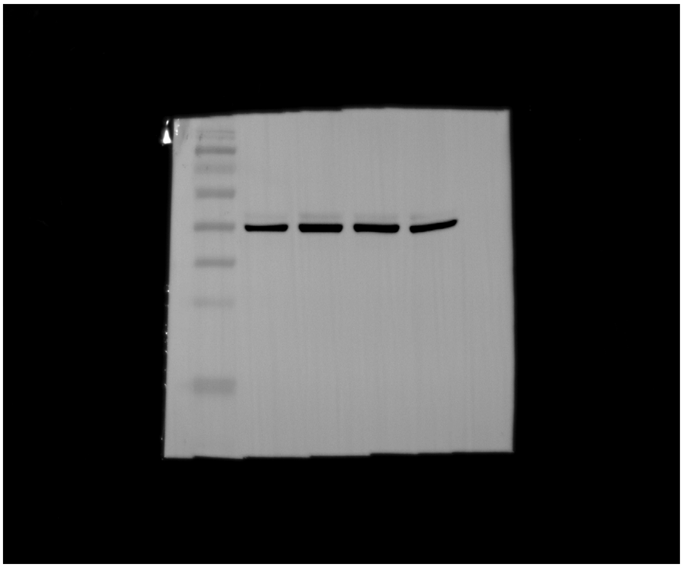

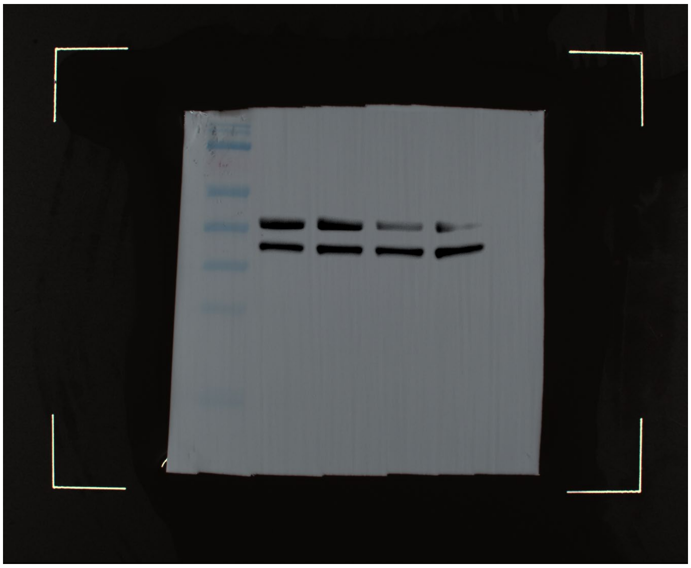

WB images for Figure 8D (HMGB1) Replicate 1

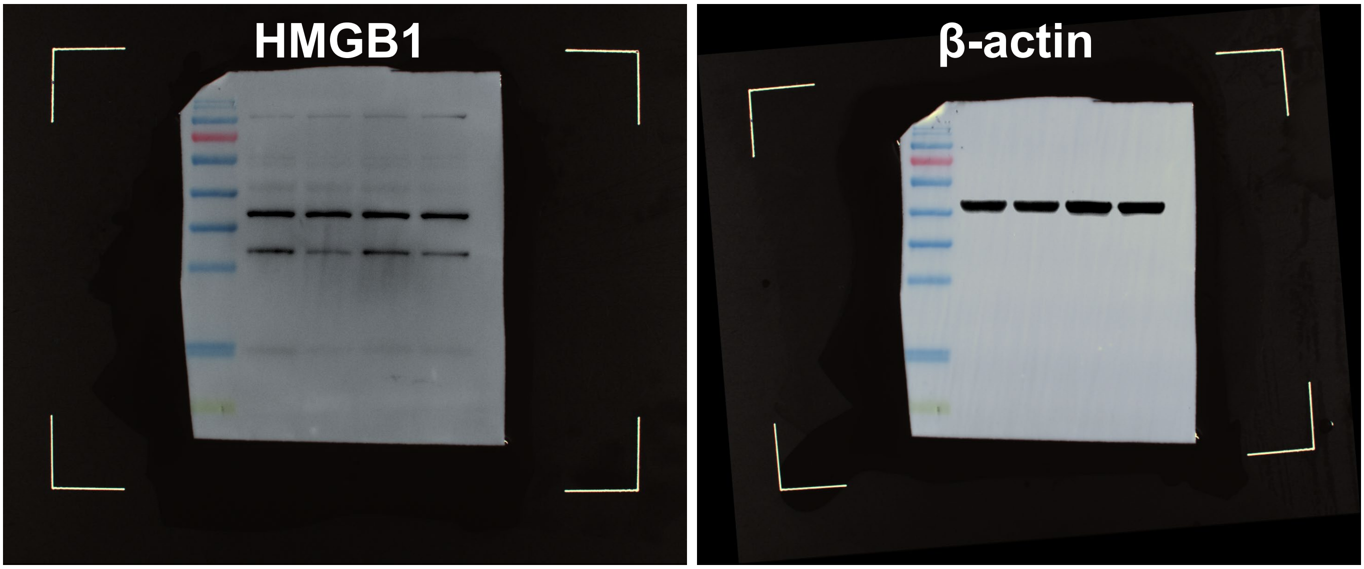


WB images for Figure 8D (HMGB1) Replicate 2

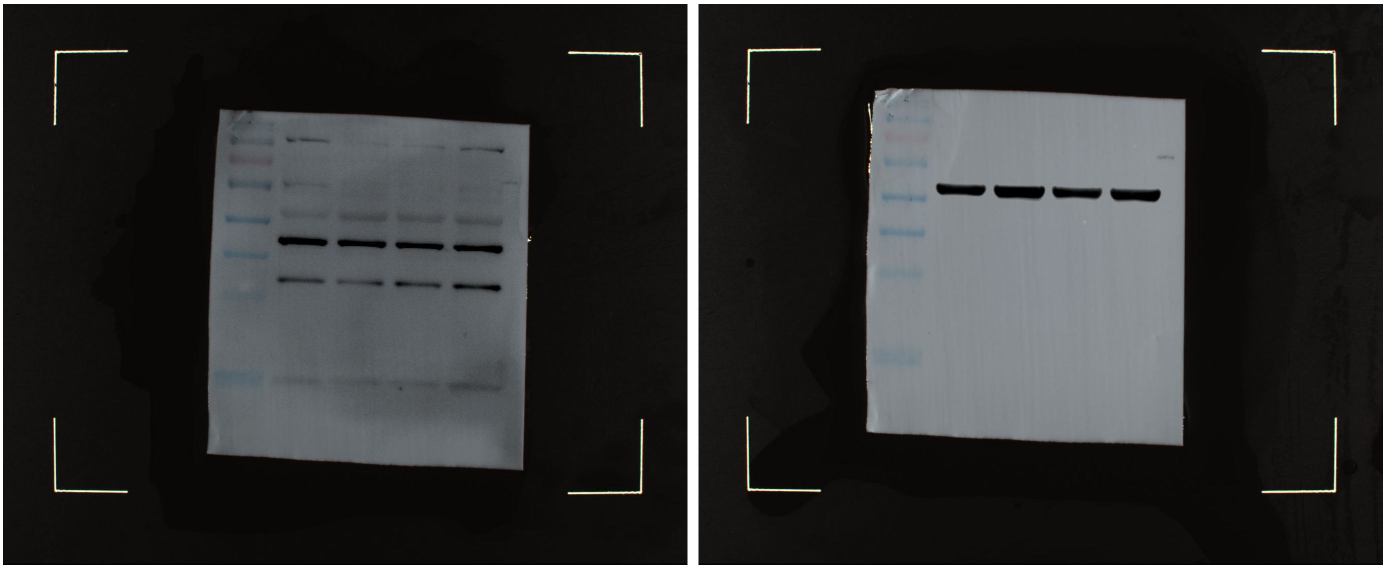

WB images for Figure 8D (HMGB1) Replicate 3

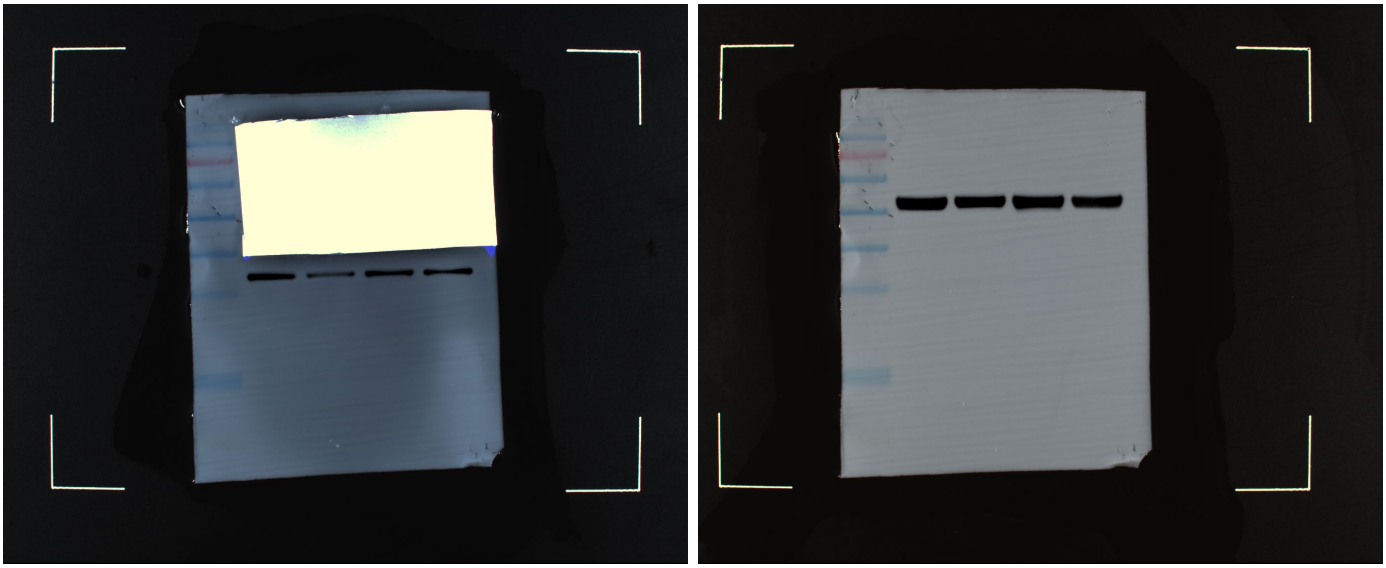

WB images for Figure 8D (HMGB1) Replicate 4

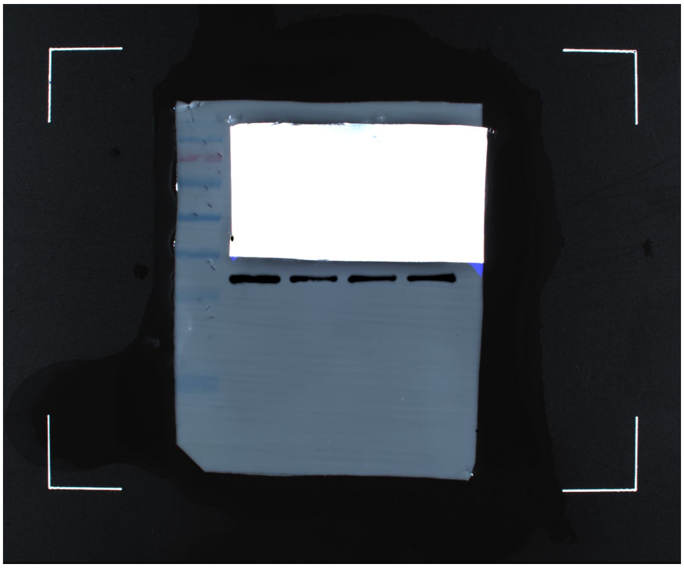

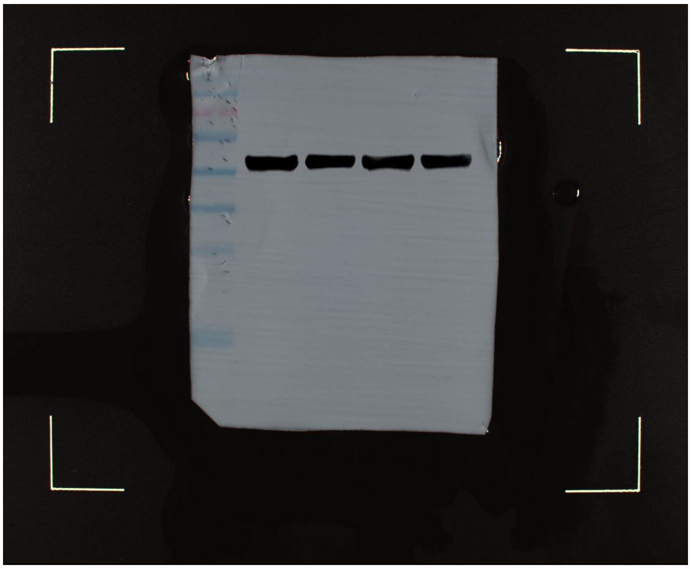

WB images for Figure 8D (HMGB1) Replicate 5

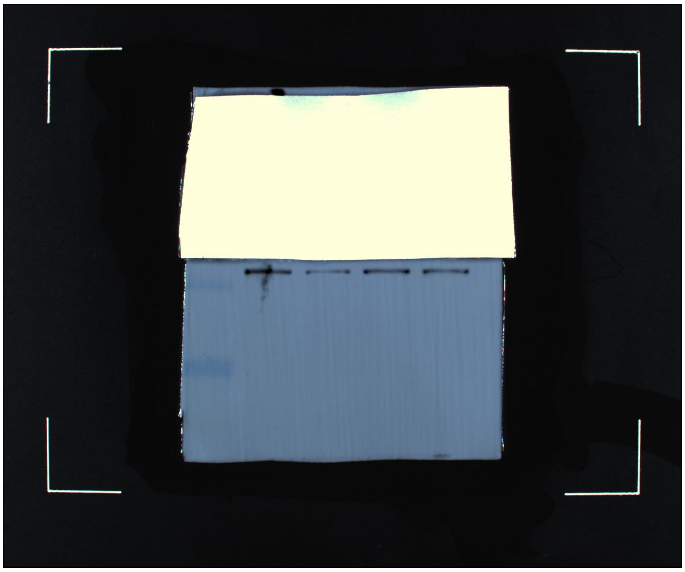

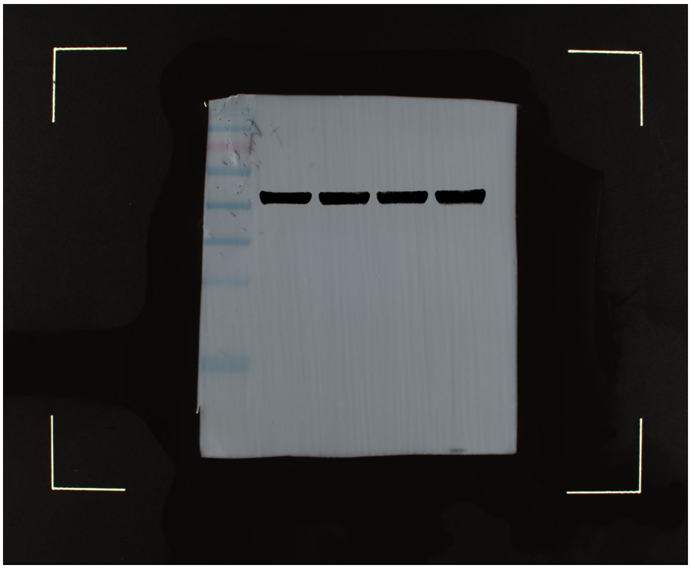

WB images for Figure 8H (HMGB1) Replicate 1

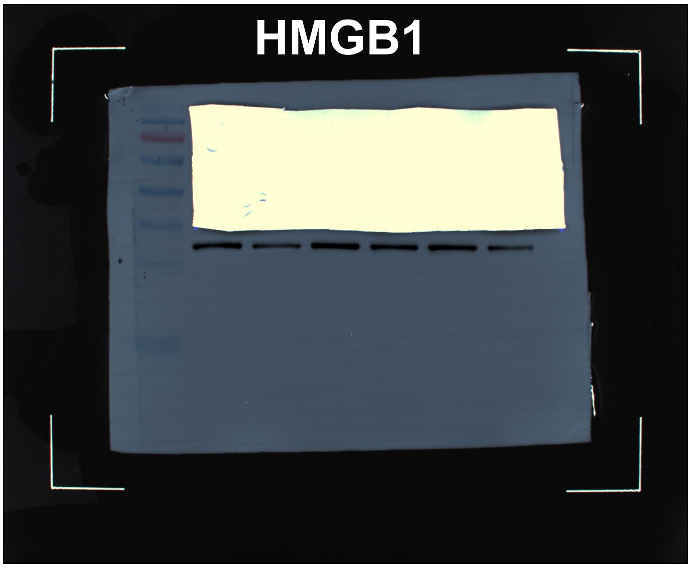


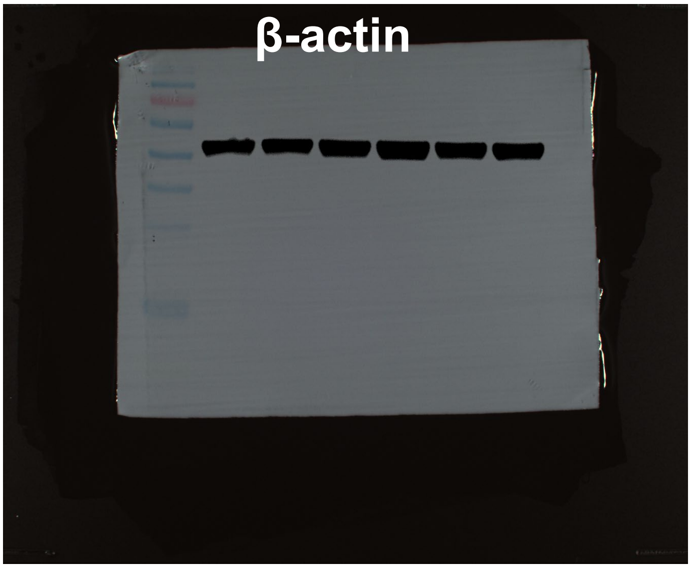

## WB images for Figure 8D (HMGB1) Replicate 2

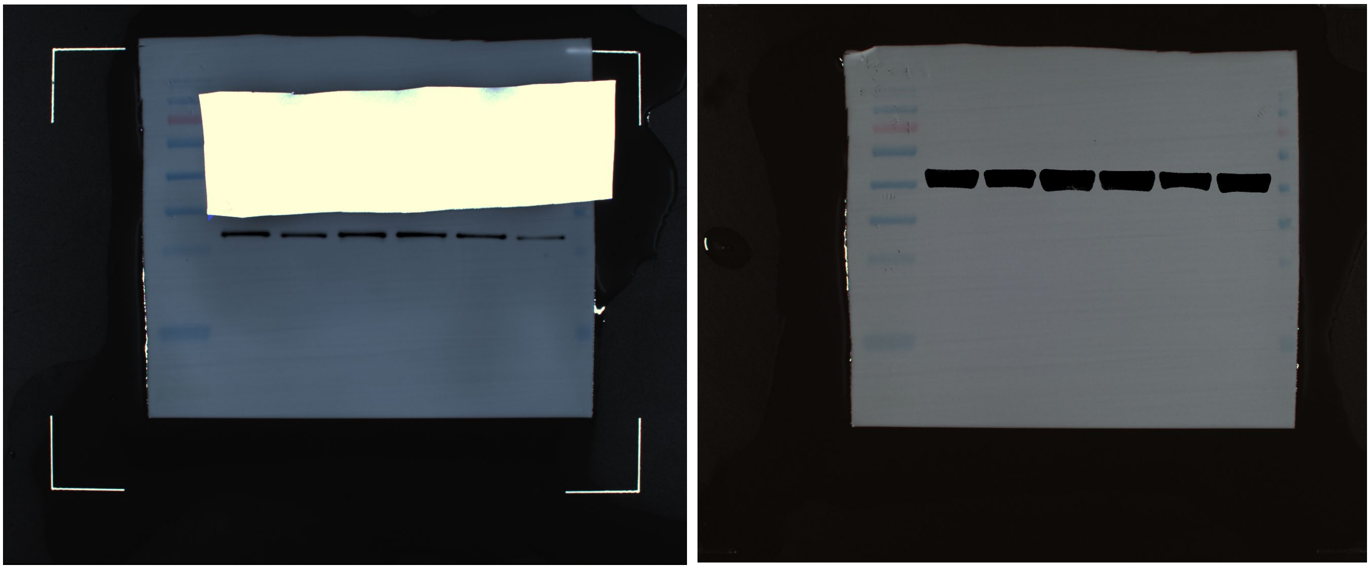

## WB images for Figure 8D (HMGB1) Replicate 3

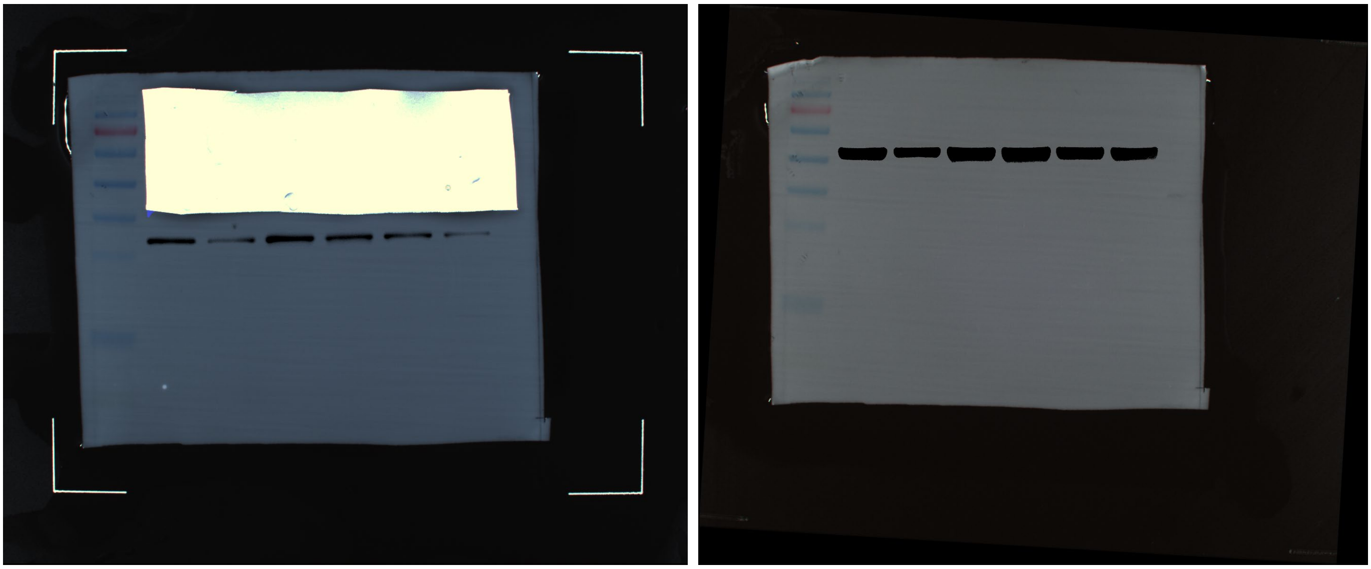

## WB images for Figure 8D (HMGB1) Replicate 4

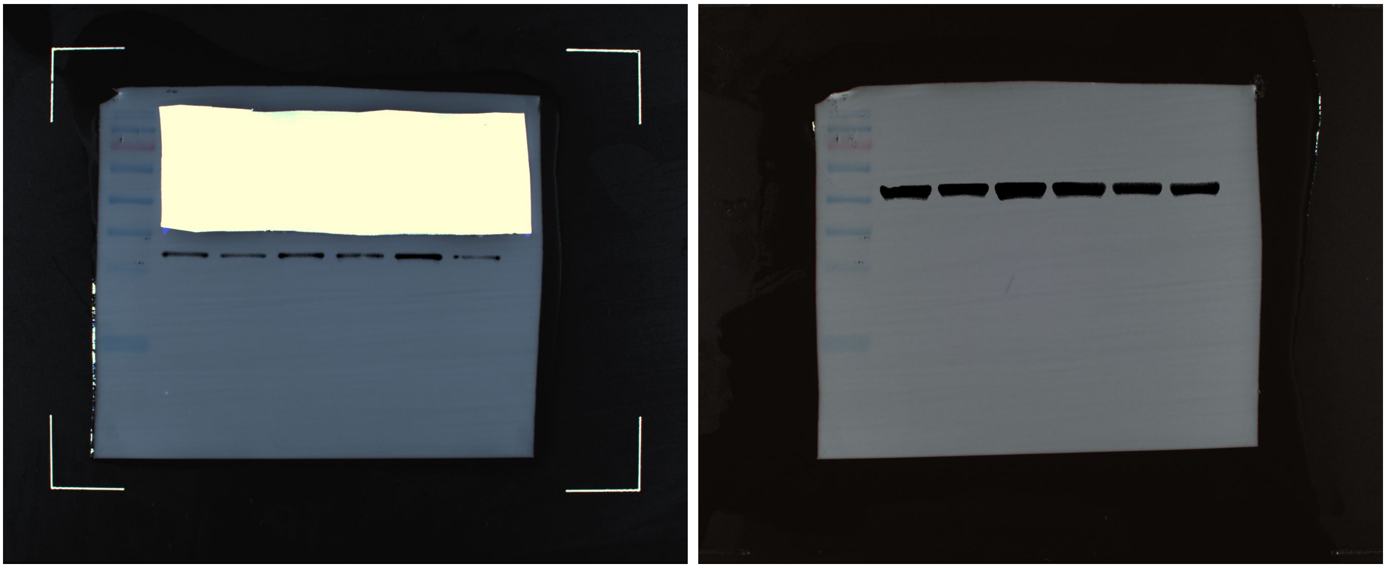

# WB images for Figure 8D (HMGB1) Replicate 5

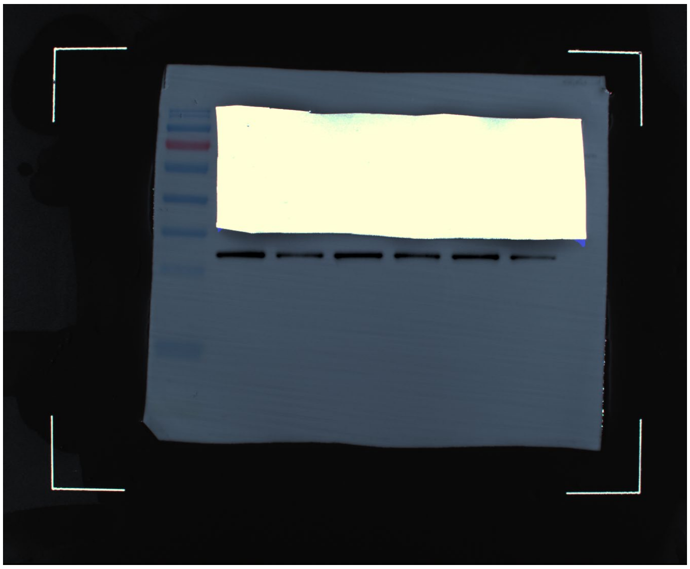

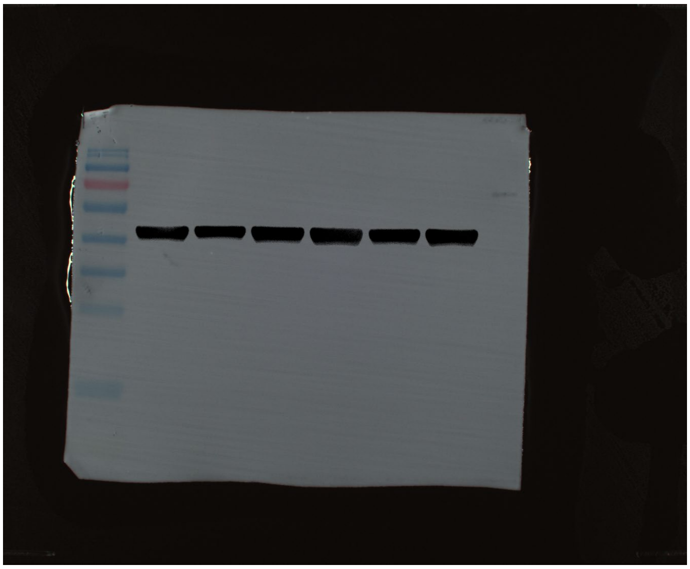

Supplement: Supplementary file 2 [file 13020_2025_1303_MOESM2_ESM.pdf]
